# The key role of the scaffold on the efficiency of dendrimer nanodrugs

**DOI:** 10.1038/ncomms8722

**Published:** 2015-07-14

**Authors:** Anne-Marie Caminade, Séverine Fruchon, Cédric-Olivier Turrin, Mary Poupot, Armelle Ouali, Alexandrine Maraval, Matteo Garzoni, Marek Maly, Victor Furer, Valeri Kovalenko, Jean-Pierre Majoral, Giovanni M. Pavan, Rémy Poupot

**Affiliations:** 1 Laboratoire de Chimie de Coordination du CNRS, UPR 8241, 205 route de Narbonne, BP 44099, 31077 Toulouse Cedex 4, France; 2 Université de Toulouse, UPS, INP, LCC, F-31077 Toulouse, France; 3 Centre de Physiopathologie de Toulouse Purpan, F-31300 Toulouse, France; 4 INSERM, U1043; CNRS, U5282; Université de Toulouse, UPS, Toulouse, France; 5 Centre de Recherche en Cancérologie de Toulouse, F-31300 Toulouse, France; 6 INSERM, U1037; CNRS, U5294; Université de Toulouse, UPS, Toulouse, France; 7 Department of Innovative Technologies, University of Applied Sciences and Arts of Southern Switzerland, Galleria 2, 6928 Manno, Switzerland; 8 Faculty of Science, J.E. Purkinje University, Ceske mladeze 8, 400 96 Ústí nad Labem, Czech Republic; 9 Kazan State Architect and Civil Engineering University, Zelenaya 1, Kazan 420043, Russia; 10 A.E. Arbuzov Institute of Organic and Physical Chemistry of Kazan Scientific Center of Russian Academy of Science, Arbuzov Str., 8, Kazan 420088, Russia

## Abstract

Dendrimers are well-defined macromolecules whose highly branched structure is reminiscent of many natural structures, such as trees, dendritic cells, neurons or the networks of kidneys and lungs. Nature has privileged such branched structures for increasing the efficiency of exchanges with the external medium; thus, the whole structure is of pivotal importance for these natural networks. On the contrary, it is generally believed that the properties of dendrimers are essentially related to their terminal groups, and that the internal structure plays the minor role of an ‘innocent' scaffold. Here we show that such an assertion is misleading, using convergent information from biological data (human monocytes activation) and all-atom molecular dynamics simulations on seven families of dendrimers (13 compounds) that we have synthesized, possessing identical terminal groups, but different internal structures. This work demonstrates that the scaffold of nanodrugs strongly influences their properties, somewhat reminiscent of the backbone of proteins.

## Abstract (web summary)

The biological properties of dendrimers are thought to be largely dependent on the chemical nature of their surface. Here, the authors show that the internal scaffold of dendritic nanodrugs strongly influences their bioactivity, based on convergent information from biology and computation results.

## Graphical abstract

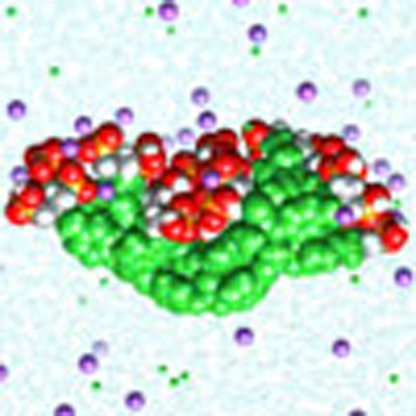

The large number of potential applications of dendrimers^1^ generates each year a tremendous amount of work, often connected to their biological properties. Emphasis is generally put on the modification of the terminal groups and of their number (related to the generation, that is, the number of layers), in connection with the multivalency effect that is the most important property recognized for dendrimers^2^,^3^,^4^,^5^. The possibility to design molecules with controlled multivalency is particularly important for biological applications^6^, polyvalent interactions being ubiquitous in many biological systems^7^. Only very few publications have experimentally reported so far the influence of the internal structure of dendrimers on their properties, even if among the five critical nanoscale design parameters recently proposed by Tomalia^8^ (size, shape, surface chemistry, flexibility and architecture), at least three of them are related to the internal structure. Comparison between PAMAM (polyamidoamine)^9^ and PPI (polypropyleneimine)^10^ dendrimers has emphasized the difference of the length of branches as the most important characteristics for their use as sensor^11^, and for obtaining nanoparticles^12^. Comparison of the physical properties have shown important differences between PAMAM and poly(L-lysine) dendrimers^13^, whereas rigid branches of dendrimers with azobenzene core induce significant differences for the isomerization, compared with less rigid branches^14^. In biology, a few examples have compared the efficiency of specific dendrimers with that of PAMAM dendrimers, with particular emphasis on transfection experiments^15^. However, there is no example to date of a study assessing the influence of a large number of dendritic scaffolds on the biological properties *per se*, and getting insights on the reasons for the differences.

Immunogenicity of dendrimers has been investigated for years, and these studies show absence or only weak immunogenicity of these molecules^16^. On the other hand, it is known that phagocytes of the immune system (that is, monocytes and macrophages that are immune white cells playing multiple roles in the immune system^17^) engulf nanoparticles, which in some cases leads to their activation^18^. Moreover, due to their involvement in many different diseases, monocytes are relevant targets to promote curative immunomodulation^19^,^20^. Some of us have already shown that a first-generation poly(phosphorhydrazone) dendrimer ended by azabis(phosphonic acid) groups has unprecedented biological properties. This compound is able to modulate *in vitro* the response of the human immune system, in particular, by inducing the multiplication of natural killer cells^21^,^22^, activating monocytes^23^ through an anti-inflammatory pathway^24^. The efficacy of this molecule has been proven *in vivo* in a mouse model of experimental arthritis relevant to human rheumatoid arthritis^25^. In this model, there is a constitutive inflammatory activation of monocytes/macrophages that is responsible for the onset and the development of the pathology. We have shown that this particular azabis(phosphonic acid)-ended dendrimer targets monocytes/macrophages and inhibits the main physiopathological features of the disease—systemic inflammation, cartilage degradation and bone resorption. The potential of this nanodrug candidate against rheumatoid arthritis has been highlighted^26^,^27^. This preliminary work has demonstrated that the N(CH_2_P(O)(OH)(ONa))_2_ pincer is the most active part within the structure. Variation on the structure of the pincer strongly decreases the biological activity^21^, whereas the replacement of phosphonic acids by carboxylic acids or sulfonic acids precludes any activity^28^. Furthermore, the azabisphosphonic pincer has to be linked to the first-generation poly(phosphorhydrazone) dendrimer (12 terminal groups) through the nitrogen atom. These poly(phosphorhydrazone) dendrimers are still very active with a lower number of such terminal functions (8 or 10), but become poorly active with 6, 4 or 2 terminal functions, and the monomer is non-active at all^29^, emphasizing the fact that these dendrimers are not drug carriers^30^, but drugs by themselves. An increased number of terminal functions (16, 24 (generation 2)^21^ or 30) has also a detrimental influence on the efficiency^29^.

Such types of terminal groups appear appealing for studying and rationalizing the influence of the nature of the scaffold on the properties of dendritic nanodrugs. Therefore, we describe the grafting of azabisphosphonic acids as terminal groups (4 to 12 functions) to a series of dendrimers having different internal structures. Seven different families of dendrimers (13 compounds) having identical terminal groups (azabisphosphonic derivatives), but different internal structures (PAMAM, PPI, poly(carbosilane)^31^, poly(L-Lysine)^32^ and three different types of phosphorus-containing dendrimers) are synthesized. Their efficiency for the activation of human monocytes is described. To identify the reasons of the original and surprising biological results obtained, the modelling of the structures of the dendrimers in aqueous solution by means of all-atom molecular dynamics (MD) simulations is carried out for obtaining high-resolution (atomistic) details of the configuration they assume in the real environment (solvated state).

## Results

### Syntheses of the dendrimers

**Figure 1:**
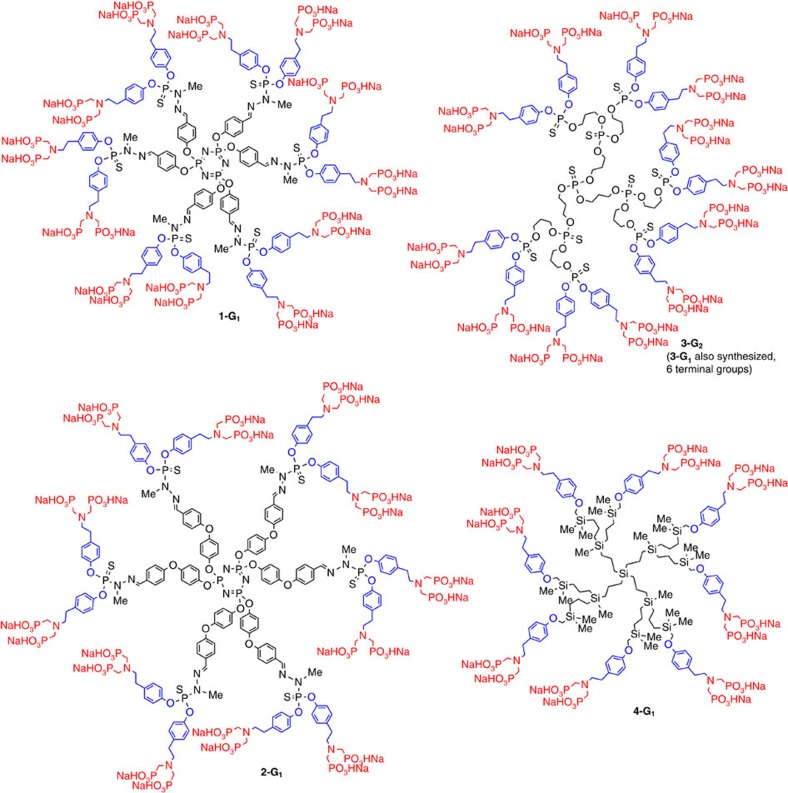
Chemical structure of dendrimers 1-G_1_–4-G_1_. The azabisphosphonic salts are in red, the linkers in blue and variable internal structures in black.

**Figure 2:**
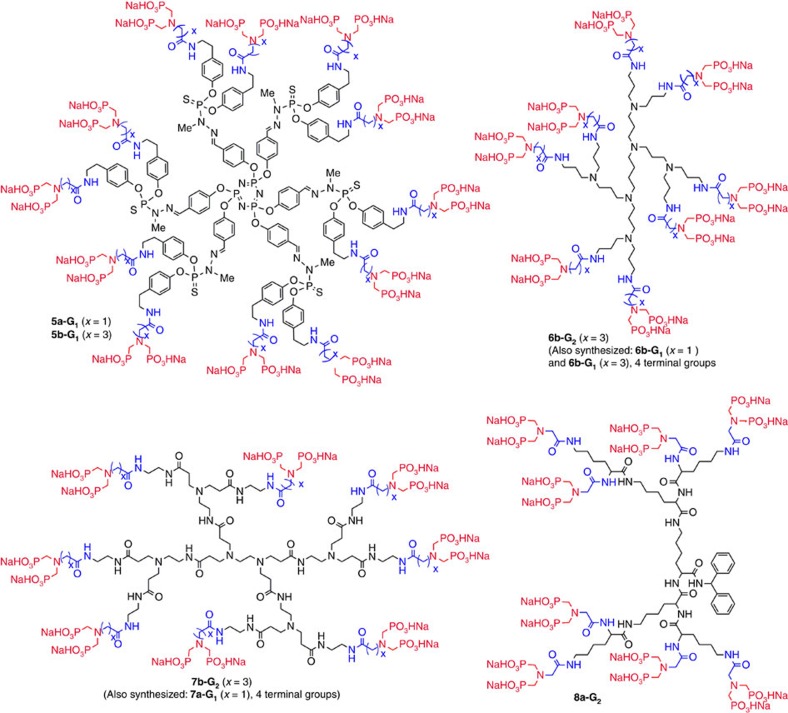
Chemical structure of dendrimers 5a,b-G_1_–8a-G_1_. The azabisphosphonic salts are in red, the linkers in blue and variable internal structures in black.

The seven different families of dendrimers (13 compounds) ended by azabisphosphonic groups that we have synthesized and tested are shown in Figs 1 and 2. Owing to the different terminal groups of the dendrimers before their functionalization by the azabis(phosphonic acid) groups, we have used two different linkers and developed different synthetic strategies.

The first linker is tyramine, which affords OC_6_H_4_CH_2_CH_2_N(CH_2_P(O)(OH)(ONa))_2_ terminal groups. In addition to the first-generation poly(phosphorhydrazone) dendrimer **1-G**_**1**_ (12 terminal functions) already synthesized^21^, this linker has been used for another type of poly(phosphorhydrazone) dendrimer having internal branches extended by an arylether linkage, **2-G**_**1**_ (12 terminal functions), as well as for poly(thiophosphate)^33^ dendrimers **3-G**_**1**_ (6 terminal functions) and **3-G**_**2**_ (12 terminal functions) and for a poly(carbosilane) dendrimer **4-G**_**1**_ (8 terminal functions; Fig. 1).

The second type of linker is obtained from the NH_2_ terminal groups of the initial dendrimers via an amide linkage (NHC(O)(CH_2_)_x_N(CH_2_P(O)(OH)(ONa))_2_ with *x*=1 or 3; Fig. 2). These terminal groups have been grafted also to the surface of the first-generation poly(phosphorhydrazone) dendrimer^34^ via the tyramine function, to afford dendrimers **5a-G**_**1**_ (*x*=1) and **5b-G**_**1**_ (*x*=3) (12 terminal functions for both). The other types of dendrimers that we have synthesized with this second type of linker are different generations of PPI dendrimers, **6a-G**_**1**_ (*x*=1) and **6b-G**_**1**_ (*x*=3; 4 terminal functions for both) and **6b-G**_**2**_ (*x*=3; 8 terminal functions); different generations of PAMAM dendrimers, **7a-G**_**1**_ (*x*=1; 4 terminal functions) and **7b-G**_**2**_ (*x*=3; 8 terminal functions); and the poly(L-lysine) dendrimer, **8a-G**_**2**_ (*x*=1; 8 terminal functions). In all cases, the azabisphosphonic terminal groups are grafted to the dendrimers in the form of the corresponding methyl ester phosphonates, to carry out the reaction in organic solvents in which both reagents are soluble.

**Figure 3:**
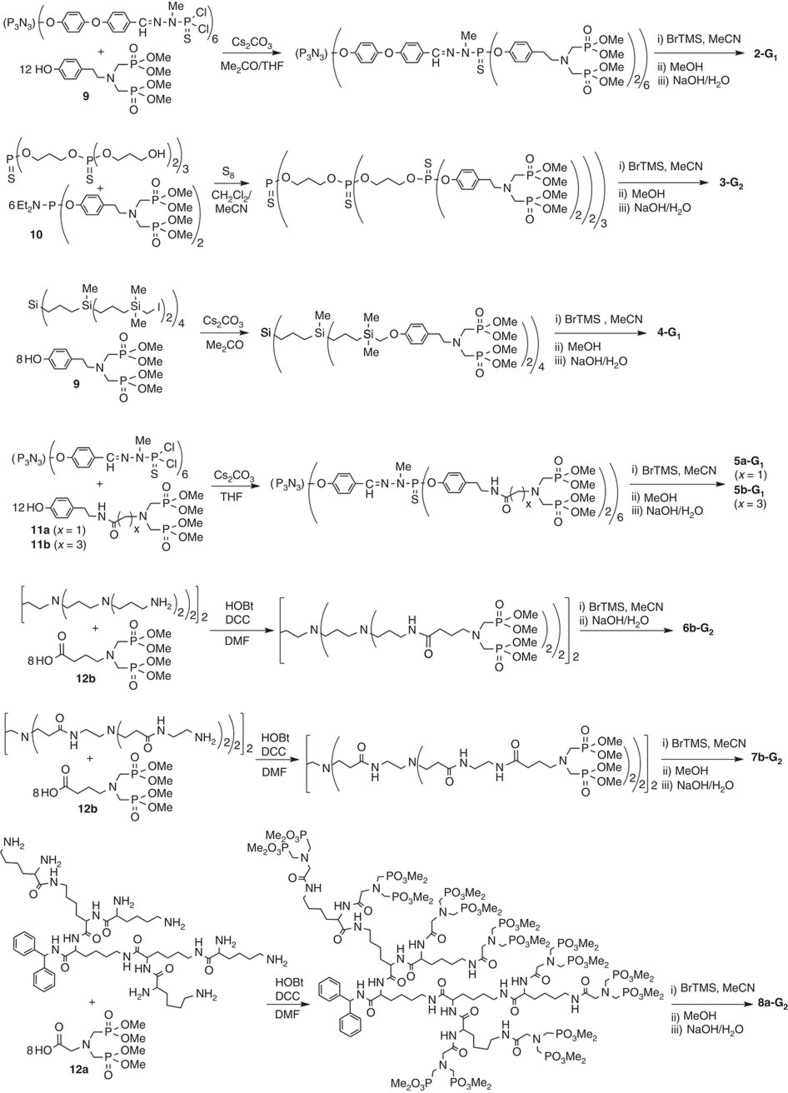
The different methods of synthesis of the dendrimers. Dendrimer **3-G**_**1**_ is synthesized as **3-G**_**2**_; dendrimers **6a-G**_**1**_ and **6b-G**_**1**_ as **6b-G**_**2**_; and dendrimers **7a-G**_**1**_ as **7b-G**_**2**_ (HOBt: hydroxybenzotriazole, DCC: *N*,*N*′-dicyclohexylcarbodiimide, BrTMS: bromotrimethylsilane).

As shown in Fig. 3, we have used different strategies for grafting the azabisphosphonates to the various types of dendrimers. The first step of the synthesis of dendrimer **2-G**_**1**_ is the nucleophilic substitution on the P(S)Cl_2_ terminal groups by the phenol moieties of the functionalized tyramine **9** in basic conditions; this method is identical to that used for the synthesis of **1-G**_**1**_ (ref. 21). The same phenol **9** was used for the nucleophilic substitution on the CH_2_I terminal groups of the carbosilane dendrimer, to finally afford **4-G**_**1**_. The phosphane bisphosphonate **10** was used for phosphitylation of the alcohol end groups of the thiophosphate dendrimers, followed by oxidation of P^III^ with sulphur, finally affording **3-G**_**1**_ or **3-G**_**2**_, depending on the generation of the starting dendrimer. The other types of tyramine derivatives **11a,b** (**a**: CH_2_ spacer, **b**: CH_2_CH_2_CH_2_ spacer) were also used for nucleophilic substitutions on P(S)Cl_2_ terminal groups, to obtain dendrimers **5a-G**_**1**_ and **5b-G**_**1**_. Compounds **11a** and **11b** were obtained by reaction of the carboxylic acids **12a** and **12b** with the NH_2_ function of tyramine via peptide coupling. In the case of dendrimers ended by NH_2_ groups (PPI, PAMAM, polylysine), this peptide coupling was carried out directly on the dendrimers with the derivatives **12a** and **12b**, affording finally the series of dendrimers **6a-G**_**1**_, **6b-G**_**1**_, **6b-G**_**2**_ (PPI-type), **7a-G**_**1**_, **7b-G**_**2**_ (PAMAM-type) and **8a-G**_**2**_ (Poly-L-lysine type).

The terminal methyl ester phosphonate groups were converted to phosphonic acid salts by reaction with bromotrimethylsilane, MeOH and then NaOH in water. Remarkably, this process induced the cleavage of the terminal phosphonic ester groups, without destroying the internal structure of the dendrimers, even in the case of dendrimers **3-G**_**1**_ and **3-G**_**2**_, which have other types of alkyl phosphonates in their internal structure.

### Monocyte activation by dendrimers

**Figure 4:**
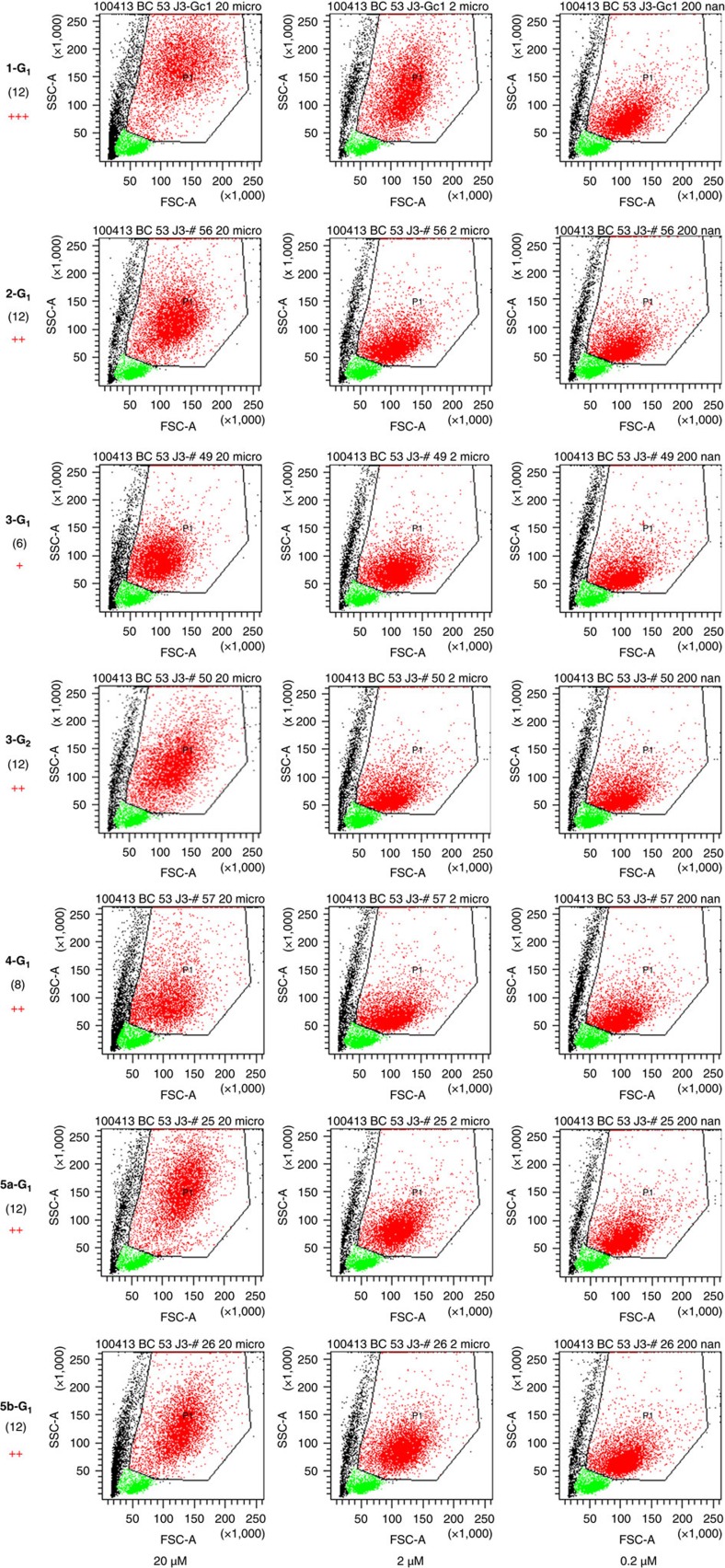
Activation of human monocytes by the series of dendrimers **1-G**_**1**_, **2-G**_**2**_, **3-G**_**1**_, **3-G**_**2**_, **4-G**_**1**_, **5a-G**_**1**_ and **5b-G**_**1**_. The bioactivity of the dendrimers is analysed by flow cytometry. Each dot in the plots is indicative of morphological change (size—the Forward Scatter (FSC) parameter on the *x* axis—and granularity—the Side Scatter (SSC) parameter on the *y* axis) undergone by purified monocytes in the presence of the different dendrimers at 20, 2, and 0.2 μM (left, middle, and right graphs respectively). Red points are monocytes (gated in the polygon), green points are remaining lymphocytes after purification, black points are died or dying cells. For each dendrimer, the number of terminal functions is indicated in parentheses. The score attributed to each dendrimer appears in red on the left, from 0 (no activation) to +++ (the highest activity, attributed to **1-G**_**1**_). Data are from one representative experiment out of six.

**Figure 5:**
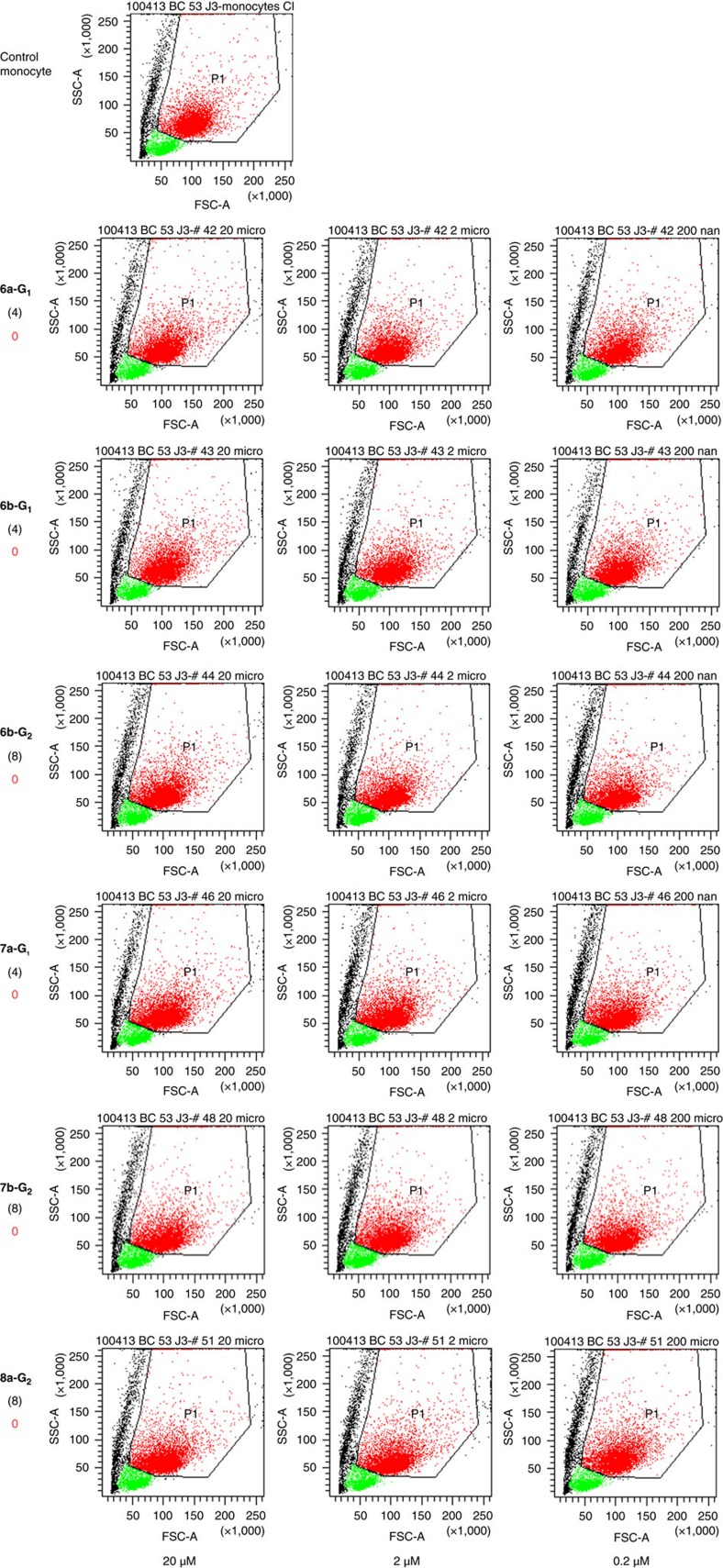
Activation of human monocytes by the series of dendrimers **6a-G**_**1**_, **6b-G**_**1**_, **6b-G**_**2**_, **7a-G**_**1**_, **7b-G**_**2**_ and **8a-G**_**2**_. The bioactivity of the dendrimers is analysed by flow cytometry. Each dot in the plots is indicative of morphological change (size—the forward scatter (FSC) parameter on the *x* axis—and granularity—the side scatter (SSC) parameter on the *y* axis) undergone by purified monocytes in the presence of the different dendrimers at 20, 2, and 0.2 μM (left, middle and right graphs, respectively). Red points are monocytes (gated in the polygon), green points are remaining lymphocytes after purification, black points are died or dying cells. For each dendrimer, the number of terminal functions is indicated in parentheses. The score attributed to each dendrimer appears in red on the left, 0 means no activation. The negative control, without any dendrimer, is given first. Data are from one representative experiment out of six.

On stimulation, monocytes/macrophages undergo morphological changes, that is, increase of their size and granularity^35^, indicative of an activated status. In particular, we have shown that phosphorus-containing anti-inflammatory dendrimers induce these morphological changes of monocytes^23^. Therefore, we have screened the biological properties of the series of dendrimers synthesized for this study by measuring the morphological features of human monocytes (Figs 4 and 5; the negative control, without any dendrimer, is given first in Fig. 5). These changes appear within a few days in *in vitro* cultures of monocytes, and can be quantified by flow cytometry. These tests were conducted at three different concentrations of dendrimer (20, 2 and 0.2 μM). The more monocytes are activated by the dendrimers, the more their size and granularity are increased. So far, dendrimer **1-G**_**1**_ appears as the most active among all the dendrimers we have tested^23^,^25^,^29^. Thus, this molecule is the standard indicator of the activation of human monocytes in this study. As the dendrimers are dissolved in pure water, the negative control of the tests consists in treating the monocytes with the accurate volume of water. The qualitative results of monocyte activation are shown in Fig. 4 for the active dendrimers and in Fig. 5 for the non-active dendrimers, with indication of the efficiency score, from +++ for the most active to 0 for the least active (non-active) dendrimers. Dendrimer **1-G**_**1**_ is the only one displaying strong activity at 2 μM, and which is still active at 0.2 μM. It appears also that dendrimers **2-G**_**1**_, **3-G**_**n**_, **4-G**_**1**_ and **5a,b-G**_**1**_ display good activity (average for **3-G**_**1**_) (Fig. 4), whereas dendrimers **6a,b-G**_**n**_, **7a,b-G**_**n**_ (*n*=1, 2) and **8a-G**_**2**_ are not active at all (Fig. 5). Dendrimers **5a-G**_**1**_ and **5b-G**_**1**_ only differ by the length of the linker between the azabisphosphonate terminations and the proximal branching points (*x*=1 versus *x*=3, Fig. 2). The bioactivity of these molecules appears to be equivalent. The same observation is made with dendrimers **6a-G**_**1**_ and **6b-G**_**1**_, which are both inactive.

### All-atom MD simulations of the dendrimers in explicit solvent

**Figure 6:**
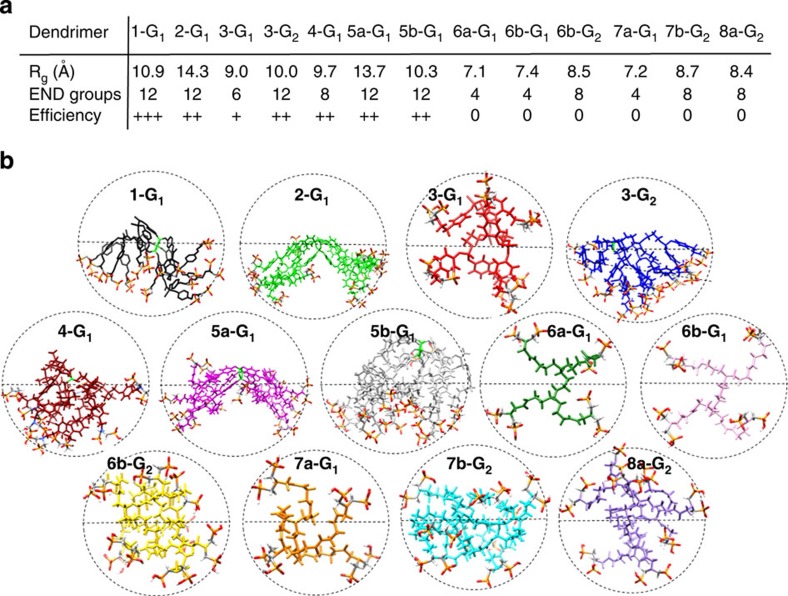
Equilibrated configurations of the 13 dendrimers and their size, obtained from the MD simulations. (**a**) Radius of gyration (*R*_g_) of the different dendrimers extracted from the equilibrated phase of the MD simulations in solution, number of azabisphosphonic surface groups (END) and biological efficiency score. Shape analysis: (**b**) MD equilibrated snapshots of the thirteen dendrimers displaying their shape. Dotted circles are added around the dendrimers to emphasize differences in the displacement of the azabisphosphonic functions around the dendrimers surface (symmetrical or directional molecules).

Molecular modelling was used to understand the striking differences observed in the biological properties. To gain molecular-level information about the dendrimers in the biological conditions, all-atom MD simulations of the 13 dendrimers in solution were carried out at 37 °C in presence of explicit water molecules and NaCl (150 mM). Each molecular system was equilibrated during 200 ns of MD simulation (Supplementary Fig. 1). Different data were extracted from the equilibrated phase MD trajectories. Figure 6a reports the equilibrated size data (that is, the radius of gyration, *R*_g_) for the dendrimers in solution. Comparison of the *R*_g_ data with the number of terminal groups indicates that neither the size (generation) of the dendrimers (we used generations 1 and 2), nor the number of terminal functions are exclusively important criteria for the biological activity of each molecule. For instance, the activity of dendrimer **4-G**_**1**_ (8 terminal functions) is marked ++, as that of dendrimers **2-G**_**1**_, **3-G**_**1**_ and **5b-G**_**1**_ (12 terminal functions); nevertheless, dendrimers **6b-G**_**2**_, **7b-G**_**2**_ and **8a-G**_**2**_ also have 8 terminal functions, but no activation properties towards monocytes.

The equilibrated snapshots taken from the MD simulations reported in Fig. 6b clearly show that the three-dimensional (3D)-geometrical arrangements of these dendrimers in the solvent are very different. Several information can be extracted from the MD simulations. Analysis of the principal moments of inertia (*I*_*x*_, *I*_*y*_ and *I*_*z*_), of the aspect ratio and of anisotropy of the equilibrated dendrimers highlight that some of them assume spherical-like rather than elongated shape in the real environment (see Supplementary Fig. 2). However, comparison of these data with the biological activity (Figs 4, 5 and 6a) demonstrates that even the overall shape of the dendrimers is not a unique discriminant parameter for their activity. The same is true for the dendrimers solvent-accessible surface area (see Supplementary Fig. 2).

However, deeper structural analysis reveals other important differences related to the location of the active surface groups in the dendrimers structure. Interestingly, as emphasized by the circle drawn around the equilibrated dendrimers in Fig. 6b, dendrimers **3-G**_**1**_, **6a,b-G**_**1**_, **6b-G**_**2**_, **7a-G**_**1**_, **7b-G**_**2**_ and **8a-G**_**2**_ are much more symmetrical than the other ones. The azabisphosphonic terminal functions are spread all over the molecular surface (sphere) for these non-active dendrimers, while, at the equilibrium, the biologically active dendrimers **1-G**_**1**_, **2-G**_**1**_, **3-G**_**2**_, **4-G**_**1**_ and **5a,b-G**_**1**_ appear as directional molecules, as in these cases the azabisphosphonic groups are gathered in half-sphere (see also Supplementary Movies 1 and 2: 1 MD 3D structure of **1-G**_**1**_, and 2 MD 3D structure of **7b-G**_**2**_). Thus, the solvated state of the different dendrimers—namely, the conformation assumed in the ‘real' environment in terms of localization and density of azabisphosphonic functions on the dendrimers surface—is morphologically very different between active and non-active dendrimers. Since the surface functionalization is identical among all dendrimers, this effect is intimately related to the different internal structure.

**Figure 7:**
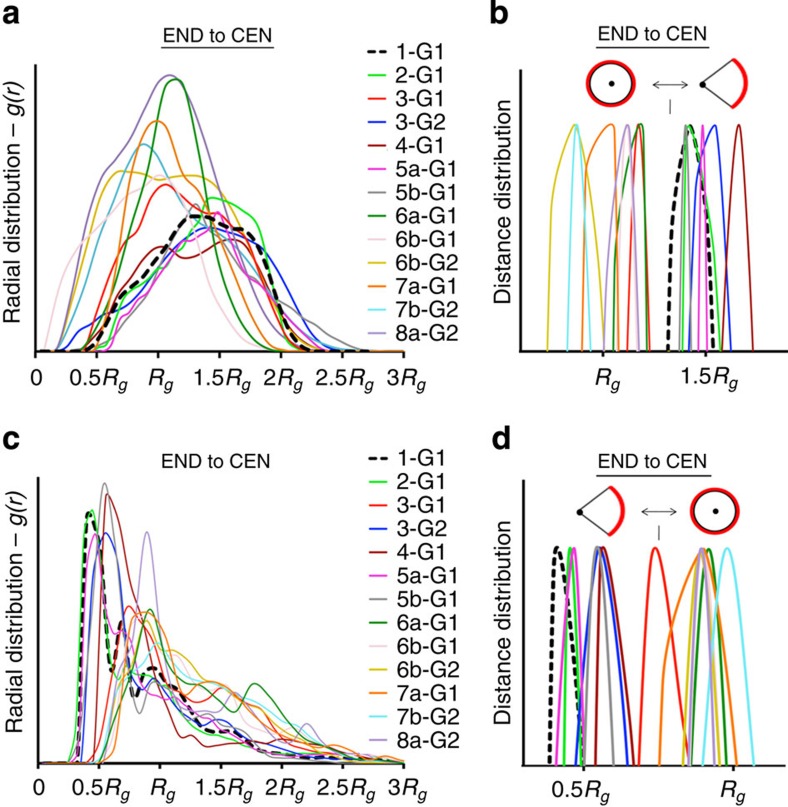
Radial distribution functions of the dendrimer terminal groups. (**a**) Radial distribution functions—*g(r)*—of the terminal groups (END) with respect to the dendrimer core unit (CEN); (**b**) normalized peaks of the *g(r)* curves (only the topmost 10%) revealing the average (most probable) END to CEN distances in the dendrimers; (**c**) radial distribution functions, *g(r)*, of the terminal groups with respect to each other; (**d**) normalized peaks of the *g(r)* curves of **c** (only the topmost 10%) revealing the average (most probable) END to END distances. In all graphs, the distance (*x* axis) is expressed in *R*_g_ units to allow comparison between different size dendrimers, and the data corresponding to dendrimer **1-G**_**1**_ is given in dotted black lines.

Further analysis on the terminal groups' location quantified these structural differences. Plots in Fig. 7a display the radial distribution functions—*g(r)*—of the azabisphosphonate terminal groups (END) calculated with respect to the core unit (CEN) of the dendrimers (Fig. 7a: END to CEN) and respect to each other (END to END: Fig. 7c). The *g(r)* curves provide indication on the relative probability to find the terminal functions at a certain distance from the centre of the dendrimer or from each other (data are averaged over the last 50 ns of the equilibrated MD trajectories). The positions of the *g(r)* maximum peaks are particularly interesting. Figure 7b,d shows the normalized peaks of the *g(r)* curves—only the topmost 10%—revealing the average (most probable) distance for the azabisphosphonic terminal groups (END) respect to the dendrimers central unit (CEN) or respect to each other. Plots in Fig. 7a,b show that, in general, the surface groups are displayed at a larger distance from the dendrimer centre for the active compounds than in the case of the inactive ones (∼1.5 *R*_g_ versus ∼*R*_g_). Moreover, *g(r)* maximum peaks related to the distance between the different surface groups (Fig. 7d) show that the azabisphosphonate terminal functions are more densely packed in the active dendrimers (the distance between the terminal branching points (N atom) is ∼0.5 *R*_g_) than in the case of the inactive dendrimers (∼*R*_g_). These data provide a picture where active dendrimers look like directional molecules with all surface functions gathered together into ‘clusters', far from the dendrimers core unit (CEN), while non-active dendrimers assume a more symmetric configuration in salt water.

**Figure 8:**
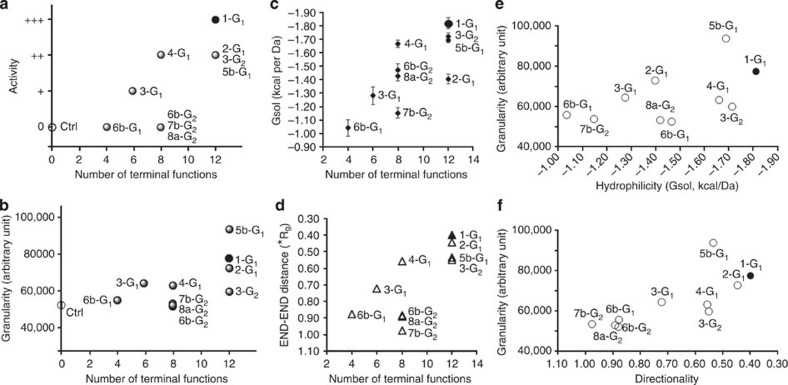
Comparison of biological activity and data from MD simulations for 10 dendrimers. (**a**) The number of terminal groups of dendrimers versus the biological efficiency. (**b**) Quantification of relative granularity (arbitrary units, means from three donors) of the monocytes treated with the dendrimers and of negative control monocytes. (**c**) *G*_sol_ values from MD (relative hydrophilicity). (**d**) Calculated distance between terminal groups (in *R*_g_ units), as an indication of molecular directionality. (**e**,**f**) Comparison between biological data and MD data: granularity versus hydrophilicity (**e**) and granularity versus directionality (**f**). The black dots correspond in all cases to the lead compound **1-G**_**1**_.

To further characterize the solvated state of the dendrimers, the solvation energy (*G*_sol_)—namely, the solute–solvent interaction energy, or the energy necessary to drag the dendrimers out from water—was also extracted from the equilibrated phase MD simulations (the last 100 ns) and used as a descriptor of molecular hydrophilicity^36^. In general, the higher (the more negative) the *G*_sol_, the more favourable the interactions of the dendrimer with water, which is a signal of overall hydrophilicity^36^. The *G*_sol_ data were further normalized for the MW of each dendrimer for comparison between different size, generation and structure of dendrimers (Fig. 8c)^36^. Additional information on the hydration of the dendrimers' interior (water penetration into the scaffold) was also extracted from the MD simulations (the last 100 ns) corroborating this analysis (see Supplementary Fig. 3).

## Discussion

As evidenced from Figs 4 and 5, the various dendrimers induced very different results for the activation of monocytes (measured by the increase of size and granularity), despite their identical terminal groups. It is also clear from Fig. 6a that the number of terminal functions is not exclusively an important criterion. As the striking differences in the properties of all these dendrimers do not seem related to the terminal groups, we hypothesized that they could be related to the internal structure. At first glance, from the chemical structure point of view, the dendrimers could be divided into two families—those having aromatic groups in their structure and those essentially constituted of alkyl linkages. All dendrimers having aromatics in their structure (**1-G**_**1**_, **2-G**_**1**_, **5b-G**_**1**_) are indeed active, but several dendrimers composed of alkyl linkages (**3-G**_**1**_, **3-G**_**2**_, **4-G**_**1**_) are also active.

In view of these puzzling biological results, theoretical calculations have been carried out to try to rationalize them. We have employed all-atom MD simulations for the 13 different dendrimers immerged in a solvation box containing explicit water molecules and salt ions to gain a molecular-level detailed description of these macromolecules in the real environment.

The radii of gyration (*R*_g_) of the equilibrated dendrimers (Fig. 6a) demonstrate that, similar to the number of terminal groups, size is not a discriminant factor controlling molecular activity. One reasonable hypothesis was that dendrimers' activity is somehow related to the shape or configuration assumed by the dendrimers in solution. However, the aspect ratio and the anisotropy parameters for the equilibrated dendrimers extracted from the MD simulations show that even the overall molecular shape is not a key parameter controlling activity (see Supplementary Fig. 2). Nevertheless, the equilibrated conformations assumed by the dendrimers in solution present other interesting differences. In particular, the structure of the active dendrimers is segregated in salt water, with all the hydrophilic terminal functions compacted on one side and the hydrophobic scaffold exposed to the external media (Fig. 6b). On the contrary, the non-active dendrimers have a more symmetrical structure with terminal groups displayed all around the dendrimer surface. Thus, our MD simulations divide the dendrimers into two categories identified by either a directional or a spherical configuration. The radial distribution functions *g(r)* data of the azabisphosphonic terminal groups (END) provide quantification for this observation (Fig. 7). In particular, the high *g(r)* peaks at short END–END distance (Fig. 7d) show that the terminal functions are more densely packed in the biologically active dendrimers, in particular for the most active **1-G**_**1**_. In general, if the azabisphosphonic terminal groups packing density (position of the END–END *g(r)* peak) is taken as a score of molecular directionality, Fig. 7c,d show that the latter is in remarkable trend with biological activity.

To better quantify the structure–activity relationships, we have assessed the biological property of the set of dendrimers towards the activation of human monocytes, as there are major players in many different diseases^19^,^20^,^25^ The biological activity of the molecules has been quantified by flow cytometry. Data shown in Figs 4 and 5 indicate that the different length of the linker (*x*=1 or *x*=3) has negligible effect within the same series of dendrimers (**5a-G**_**1**_/**5b-G**_**1**_ and **6a-G**_**1**_/**6b-G**_**1**_). A similar comment can be done also on the results from MD, both in terms of 3D geometrical arrangement of the molecules and of quantification of the structural differences (Figs 6 and 7). Therefore, we discarded the three molecules **5a-G**_**1**_, **6a-G**_**1**_ and **7a-G**_**1**_ from the initial set of 13. Figure 8a displays the biological activity of the 10 remaining dendrimers, as a function of the number of terminal groups. As taken alone the cell size parameter is poorly discriminating between the dendrimers, we have quantified and used the relative granularity of monocytes (at the highest concentration of dendrimers, that is, 20 μM; Fig. 8b): the more granulous the monocytes, the more active the dendrimer.

For better understanding the key parameters of the dendrimers structure for the biological activity, several other information have been extracted from the MD data. The *G*_sol_ energies extracted from the equilibrated phase (the last 100 ns) MD simulations of the dendrimers in solution are representative of the level of hydrophilicity/hydrophobicity of their solvated state. Figure 8c shows that also *G*_sol_ data are in good trend with the molecular activity of the dendrimers (Fig. 8a), which suggests that both hydrophilicity/hydrophobicity (Fig. 8c), and molecular directionality (Fig. 8d) are key discriminant parameters for biological activity (Fig. 8a). In fact, the most active dendrimers, with particular emphasis on dendrimer **1-G**_**1**_, possess the most favourable solvation energy (*G*_sol_) and the highest directionality scores (the smaller the END–END distance, the higher molecular directionality), which also points towards a direct correlation between dendrimers multivalency and activity. In fact, at the molecular level, a higher density of active functions can impact biological efficiency because it intuitively amplifies cooperativity and favours multivalent interactions. As last, we put in direct correlation biological activity (granularity) with hydrophilicity (*G*_sol_) and molecular directionality. The results are shown in Fig. 8e,f, respectively, showing remarkable trends. Indeed, looking at these dendrimers in solution, these analyses suggest that the dense concentration of active functions in regions of the dendrimers surface and the level of hydrophilicity/hydrophobicity of the structure are crucial to molecular activity. This is the case also of biological molecules like proteins, where the features of the surface, presence of charged or active patches, and overall and local hydrophobicity and hydrophilicity levels produce remarkable effects through a delicate network of multiscale interactions and molecular recognition.

In conclusion, this work carried out on seven different families of dendrimers clearly demonstrates that the internal structure of dendrimers cannot be anymore regarded as an ‘innocent' support for active functions, but plays a crucial role, especially when considering biological properties. Indeed, the geometry of the dendrimers with identical terminal groups may be very different intrinsically, and the differences can be amplified in a real environment such as a water solution or biological materials (like blood, biological barriers and cell membranes), depending on the nature of the scaffold. Structural differences induce changes in shape and in the distribution of the terminal active functions of the dendrimers, which are responsible for the biological activity. Regarding monocyte activation, the most active dendrimers are those in which the surface functions are gathered on one side (directional molecules), a suitable orientation to maximize multivalent interactions with cells. Definitely, this work shows how changing one single parameter, even in the internal moieties, may totally modify the properties of dendrimers. Therefore, the implementation of extensive MD simulation studies of dendrimers appears as a pivotal asset when designing new bio-oriented dendritic devices or to optimize existing dendritic systems. Such finding exceeds the field of dendrimers and embraces those more general of macromolecules and nanostructures. The direct relationships traced here between hydrophobicity/hydrophilicity and activity, and between activity and surface characteristics (that is, directionality in the localization of functional groups) recall the main features of biological macromolecules such as proteins. Our results are reminiscent to what is known about the interplay between natural networks and their surroundings.

## Methods

### General information about the synthesis of dendrimers

All manipulations are performed under argon using standard Schlenk techniques. Commercial samples were used as received from Aldrich (PAMAM dendrimers, DAB dendrimers, Lysine dendrimer and all other chemicals). The following compounds have been prepared according to published procedures: tyramine azabisphosphonate **9** (ref. 21) and dendrimer **1-G**_**1**_ (ref. 21), Salamonczyk's thiophosphite dendrimers^33^, chlorine-ended carbosilane dendrimers^37^ and phosphorhydrazone dendrimers ended by 12 chlorine atoms^38^. All solvents were dried and distilled according to routine procedure before use. Thin-layer chromatography was carried out on Merck Kieselgel 60F254 precoated silicagel plates. Preparative chromatography was performed on Merck Kieselgel. ^1^H, ^13^C, ^31^P NMR, HMQC and HMBC measurements were performed on Bruker AC200, AM250 and AV300 and AMX400. All dendrimers have been characterized by ^1^H, ^13^C{^1^H} and ^31^P{^1^H} NMR, and two-dimensional NMR spectra when necessary. All details of the synthesis and the NMR data for all compounds are given in the Supplementary Information. The experimental information for the synthesis of all dendrimers is also given in the Supplementary Methods for chemistry. The synthesis of dendrimer **5a-G**_**1**_ is given below as a typical example.

### Typical example for the synthesis of dendrimers

Synthesis of **5a-G**_**1**_, *first step*: 0.017 mmol of generation 1 phosphorus-containing dendrimer (12 Cl terminations)^38^ are placed in solution in 3 ml of dry tetrahydrofuran. Cs_2_CO_3_ (5.04 mmol), then 0.23 mmol of the tyramine azabisphosphonate **11a** (*x*=1) in solution in 3 ml of dry tetrahydrofuran are successively added to this solution. The mixture is stirred overnight at room temperature and then filtered on celite. The reaction medium is evaporated under reduced pressure then the dry residue is dissolved in a minimum volume of dichloromethane. The product is then precipitated in a large volume of ether. This operation is repeated three times to eliminate the slight excess of starting tyramine derivative. The dendrimer with methylphosphonate terminations is obtained as an off-white powder (88% yield). *Second step*: 0.015 mmol of dendrimer with methylphosphonate terminations are placed in solution under an inert atmosphere in 3 ml of distilled acetonitrile. The solution is taken to 0 °C then 48 equivalents of BrTMS (0.73 mmol) are added dropwise under argon. The mixture is stirred for 30 min at 0 °C, then overnight at room temperature and finally evaporated to dryness under reduced pressure. The residue thus obtained is treated with methanol (2 × 15 ml), then evaporated to dryness and washed with dry ether (20 ml) to afford the dendrimer with phosphonic acid terminations (63% yield). *Third step*: the sodium salt is obtained by reaction of 24 equivalents of NaOH solution at 0.1955 N on one equivalent of dendrimer with phosphonic acid terminations, to produce, after stirring 1 h at room temperature and freeze drying, the expected dendrimer **5a-G**_**1**_ (70% yield).

### Purification and activation of human monocytes

Preparation of PBMC (peripheral blood mononuclear cells) from healthy volunteers and subsequent purification of monocytes are performed as already described^24^. Purity of the monocytes (>95%) is assessed by flow cytometry on a LSR-II device (BD Biosciences, San Diego, CA, USA).

For activation cultures, purified monocytes are cultured in multi-well plates for 72 h at 10^6^ cells ml^−1^ of complete RPMI 1640 medium (Roswell Park Memorial Institute medium), that is, supplemented with 10% of heat-inactivated fetal calf serum, 1 mM sodium pyruvate, 2 mM L-glutamine (all from Invitrogen Corporation, Paisley, UK), penicillin and streptomycin, both at 100 U ml^−1^ (Cambrex Bio Science). Dendrimers are added at the beginning of the cultures at 20, 2 and 0.2 μM.

### Flow cytometry analyses

At the end of the culture, monocytes are washed with PBS with 5% fetal calf serum and their morphology (size and granularity) is analysed by flow cytometry (LSR-II device from BD Biosciences) and quantified using FACSDiva software (BD Biosciences). The most activated monocytes are the most granular, and biggest they appear. A score (from 0 to +++) is attributed to each dendrimer on these morphological criteria. A score of 0 is given to dendrimers that do not induce morphological changes (monocytes with the same morphology than control, non-activated, monocytes). A score of +++ is given to dendrimer **1-G**_**1**_, the most active one which induces dramatic morphological changes at 20 and 2 μM, still detectable at 0.2 μM. A score of ++ is given to dendrimers whose effect is comparable to the one of dendrimer **1-G**_**1**_ at 20 μM but with no or weak detectable effect at 2 μM. A score of + is given to dendrimers which has a detectable effect, although weak, at 20 μM.

### All-atom MD simulations

The MD simulation work is conducted by using the AMBER 12 software^39^. The molecular models for all dendrimers are created and parametrized according to a validated procedure for the simulation of dendrimers in aqueous solution (see Supplementary Table 1 for details)^36^,^40^,^41^. The force field parameters for the **4-G**_**1**_ carbosilane dendrimer are obtained as previously reported^42^. All the dendrimer models are immerged in a periodic box containing explicit TIP3P water molecules^43^ and 150 mM of NaCl to reproduce the experimental conditions. All systems are simulated for 200 ns in NPT conditions at 37 °C. Analyses of the structural features and of the solvation energies (*G*_sol_) of the dendrimers are performed on the equilibrated phase MD trajectories (Supplementary Fig. 1). Details on the computational procedures are given in the Supplementary Methods and Supplementary Movies 1 and 2 obtained from the MD simulations showing the 3D structures of **1-G**_**1**_ (MD 3-D structure of **1-G**_**1**_) and **7b-G**_**2**_ (MD 3D structure of **7b-G**_**2**_) as representative cases of directional and symmetrical molecules.

## Additional information

**How to cite this article:** Caminade, A.-M. *et al.* The key role of the scaffold on the efficiency of dendrimer nanodrugs. *Nat. Commun.* 6:7722 doi: 10.1038/ncomms8722 (2015).

## Supplementary Material

Supplementary Figures, Table, Methods and References Supplementary Figures 1-3, Supplementary Table 1, Supplementary Methods and Supplementary References

Supplementary Movie 1 Molecular dynamics movie showing the structure of the biologically active dendrimer 1-G1 in water.

Supplementary Movie 2 Molecular dynamics movie showing the structure of the biologically non-active dendrimer 7b-G1 in water.
