# Supplementary material for: The key role of the scaffold on the efficiency of dendrimer nanodrugs
Source: Nat Commun. 2015 Jul 14;6:7722. doi: 10.1038/ncomms8722 (PMC4510975; doi:10.1038/ncomms8722)
Supplement: Supplementary Figures, Table, Methods and References — Supplementary Figures 1-3, Supplementary Table 1, Supplementary Methods and Supplementary References [file ncomms8722-s1.pdf]

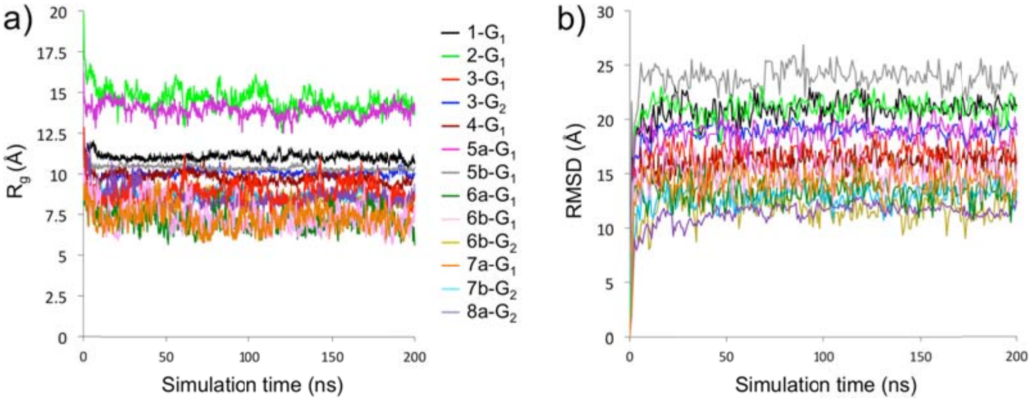

Supplementary Figure 1. Equilibration of the simulated molecular systems. (a) Radius of gyration (R_g) and (b) root mean square displacement (RMSD) data obtained from the MD simulations.

a)

Dend	1-G_1	2-G_1	3-G_1	3-G_2	4-G_1	5a-G_1	5b-G_1	6a-G_1	6b-G_1	6b-G_2	7a-G_1	7b-G_2	8a-G_2
R_g (Å)	10.9	14.3	9	10	9.7	13.7	10.3	7.1	7.4	8.5	7.2	8.7	8.4

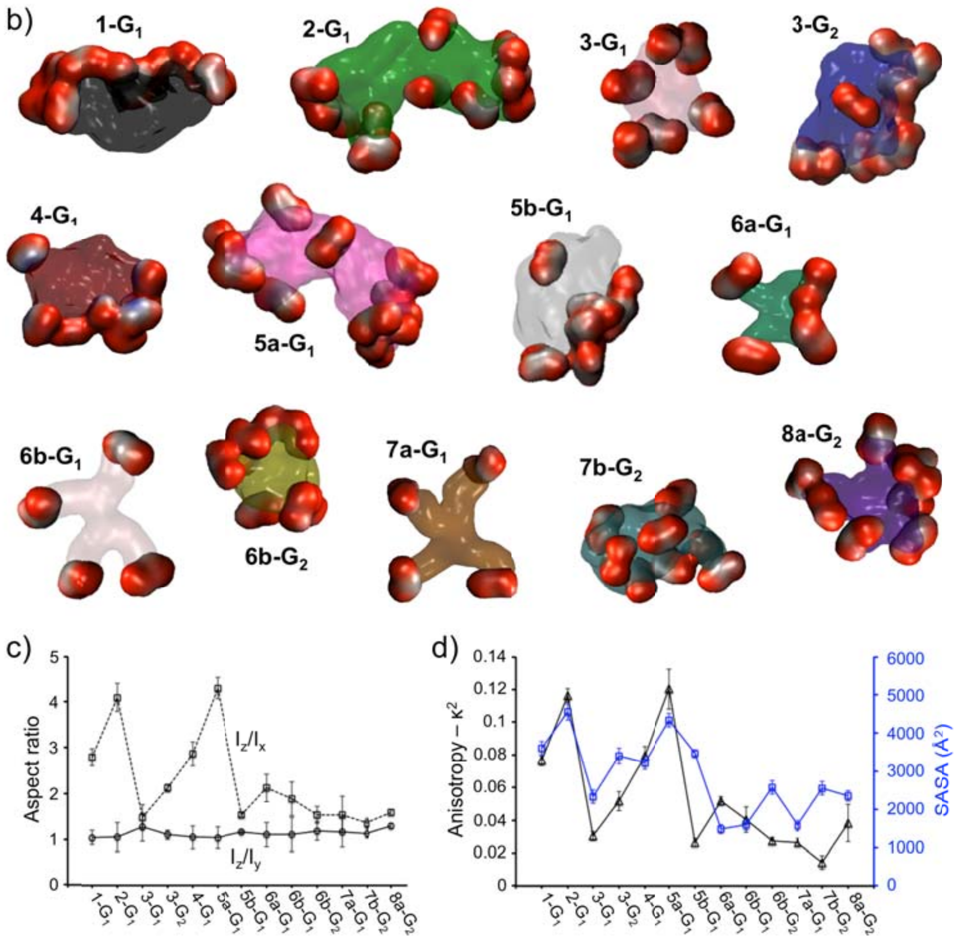

Supplementary Figure 2. Additional structural data from the MD simulations. (a) Average size (R_g) of the dendrimers in solution. (b) Equilibrated snapshots of the surface of

the dendrimers. (c) Shape: aspect ratio of the equilibrated dendrimers (ratios between the principal moments of inertia). (d) Anisotropy index (black) and solvent accessible surface area (SASA) for the equilibrated dendrimers in solution.

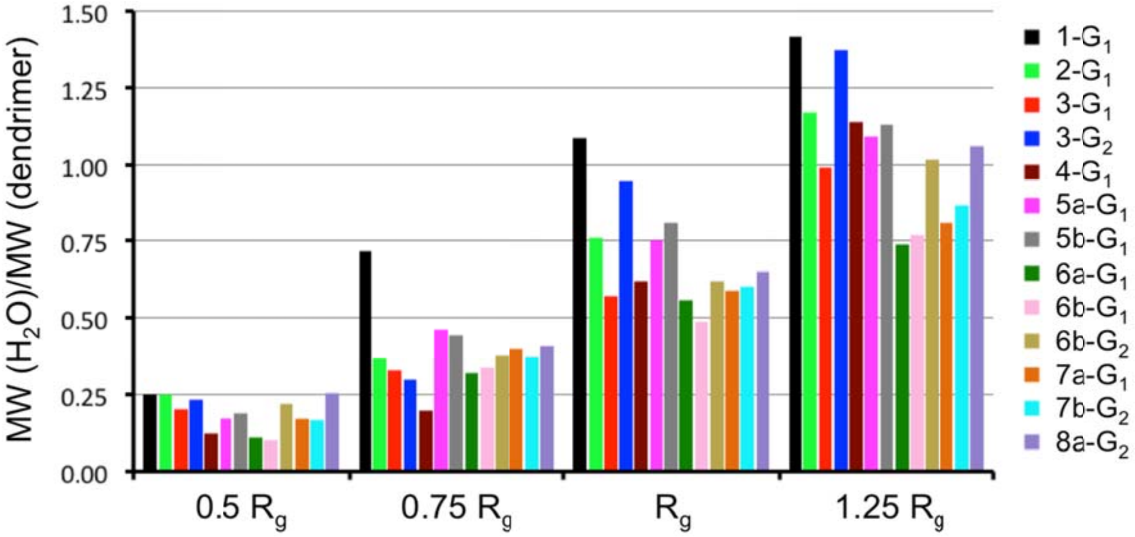

Supplementary Figure 3. Water penetration inside the dendrimers scaffold. Data are representative of the water weight fraction over the dendrimer's mass as a function of the distance (in R_g units) from the center of the dendrimer. This data is representative of the quantity of water that each dendrimer can incorporate, and thus of the level of hydration of the dendrimers.

Supplementary Table 1. Main features of the molecular systems simulated in this study.

Molecular system	Simulation temperature (°C)	Number of surface groups	MW (kDa)	Box volume ($Å^3$)	Number of Cl^- and Na+ ions in the system[a]	Number of water molecules in the system	Total number of atom in the system	Simulation time for each MD run (ns)

**1-G_1**	37	12	5.268	293609	28	9376	28773	200
**2-G_1**	37	12	5.821	398425	36	12756	38944	200
**3-G_1**	37	6	2.407	205791	60	19878	20161	200
**3-G_2**	37	12	5.163	332659	32	10674	32576	200
**4-G_1**	37	8	3.860	262144	20	6598	20343	200
**5a-G1**	37	12	5.953	405777	38	12976	39624	200
**5b-G1**	37	12	6.289	408973	38	13057	40004	200
**6a-G2**	37	4	1.289	130137	12	4178	12704	200
**6b-G1**	37	4	1.401	148978	14	4786	14556	200
**6b-G_2**	37	8	2.942	146853	14	4669	14479	200
**7a-G1**	37	4	1.489	151679	14	4887	14857	200
**7b-G_2**	37	8	3.599	257664	24	8330	25383	200
**8a-G_2**	37	8	3.025	158417	14	5065	15591	200

- [a] The suitable number of Cl^- and Na+ counter-ions is added in the system to guarantee overall neutrality and to reproduce the experimental ionic strength of 150 mM [NaCl].

Supplementary Methods for Chemistry

General procedure

All reactions were carried out in the absence of air using standard Schlenk techniques and vacuum-line manipulations when carried out in organic solvents. Commercial samples were used as received from Aldrich (PAMAM dendrimers, DAB dendrimers, Lysine dendrimer, and all other chemicals). All solvents were dried and distilled according to routine procedure before use. Thin layer chromatography was carried out on Merck Kieselgel 60F254 precoated silicagel plates. Preparative chromatography was performed on Merck Kieselgel. ^{1}H, ^{13}C, ^{31}P NMR, HMQC and HMBC measurement were performed on Bruker AC200, AM250 and AV300 and AMX400. For ^{13}C NMR spectra, the uncorrected baseline might show a deviation centred at 115 ppm due to polymer coating on the measuring apparatus. Coupling constants are reported in Hz and chemical shifts in ppm/TMS for ^{1}H and {^{1}H}^{13}C NMR and in ppm/$CFCl_3$ for {^{1}H}^{19}F NMR. Chemical shifts for ^{31}P NMR spectra are calibrated with phosphoric acid as an external reference (Bruker AC200 operating at 81.0 MHz, Bruker AM250 operating at 101.2 MHz) or with phosphoric acid as an internal reference (Bruker AV300 operating at 121.5 MHz). Residual solvent peaks are starred. The first-order peak patterns are indicated as s (singulet), d (doublet), t (triplet), q (quadruplet), qn (quintuplet). Complex non-first-order signals are indicated as m (multiplet). ^{13}C NMR signals were assigned using HMQC and HMBC sequences when required. The following compounds have been prepared according to published procedures: tyramine aza-bisphosphonate **9** and dendrimer **1-G$_1$**[1], Salamonczyk's thiophosphite dendrimers[2], Chlorine-ended carbosilane dendrimers[3], phosphorhydrazone (PPH) dendrimers ended by 12 chlorine atoms[4].

Synthesis and characterization of dendrimer 2-G$_1$

First step

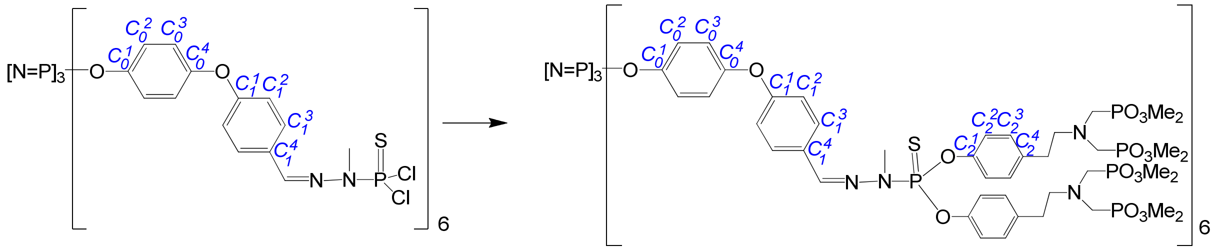

Tyramine aza-bisphosphonate **9** (388 mg, 1.020 mmol) and cesium carbonate (565 mg, 1.734 mmol) are added to a solution of the first generation aryl ether dendrimer with $PSCl_2$ terminations (177 mg, 0.074 mmol) in a mixture of acetone/THF (10 mL/3 mL). The reaction mixture is stirred at room temperature during 12 h, centrifuged and the resulting clear solution is evaporated to dryness under reduced pressure. The obtained oil is purified by chromatography on silica gel (gradient acetone/triethylamine (100:0 to 90:10), Rf = 0.97 in acetone/triethylamine (90:10)) to afford the dendrimer with dimethylphosphonate ends as a pale yellow solid (yield: 91%).

^{31}P-{^{1}H} NMR ($CDCl_3$, 121.5 MHz): δ = 9.27 (s, N_3P_3); 26.78 (s, PO_3Me_2); 63.18 (s, P_1); ^{1}H NMR ($CDCl_3$, 300.13 MHz): δ = 2.74 (t, $^{3}J_{HH}$ = 7.2 Hz, 24H, $\underline{CH_2}$-CH_2-N); 3.05 (t, $^{3}J_{HH}$ = 7.2 Hz, 24H, CH_2-$\underline{CH_2}$-N); 3.17 (d, $^{2}J_{HP}$ = 9.3 Hz, 48H, N-CH_2-P); 3.29 (d, $^{3}J_{HP}$ = 10.2 Hz, 18H, CH_3-N-P_1); 3.71 (d, $^{3}J_{HP}$ = 10.5 Hz, 77H, OMe); 3.72 (d, $^{3}J_{HP}$ = 10.5 Hz, 77H, OMe); 6.93 (m, 12H, C_0^3-H); 6.94 (m, 12H, C_1^2-H); 7.00 (m, 12H, C_0^2-H); 7.10 (m, 24H, C_2^2-H); 7.16 (m, 24H, C_2^3-H); 7.60 (br s, 6H, CH=N); 7.67 (m, 12H, C_1^3-H); ^{13}C-{^{1}H} NMR ($CDCl_3$, 75.6 MHz): δ = 32.87 (br s, CH_3-N-P_1); 33.04 (s, $\underline{CH_2}$-CH_2-N); 49.48 (dd, $^{1}J_{CP}$ = 157.5 Hz, $^{3}J_{CP}$ = 7.3 Hz, N-CH_2-P); 52.62 (d, $^{2}J_{CP}$ = 3.4 Hz, OMe); 52.66 (d, $^{2}J_{CP}$ = 3.4 Hz, OMe) ; 58.08 (t, $^{3}J_{CP}$ = 7.5 Hz, CH_2-$\underline{CH_2}$-N); 118.50 (s, C_1^2); 120.14 (s, C_0^3); 121.27 (d, $^{3}J_{CP}$ = 4.5 Hz, C_2^2); 122.23 (s, C_0^2); 128.61 (s, C_1^3); 129.87 (s, C_2^3); 130.21 (s, C_1^4); 136.47 (d, $^{5}J_{CP}$ = 1.8 Hz, C_2^4); 138.89 (d, $^{3}J_{CP}$ = 13.8 Hz, CH=N); 146.37 (td, $^{2}J_{CP}$ = 5.2 Hz, $^{4}J_{CP}$ = 2.5 Hz, C_0^1); 148.96 (d, $^{2}J_{CP}$ = 7.0 Hz, C_2^1); 153.58 (s, C_0^4); 158.32 (s, C_1^1) ppm.

Second step

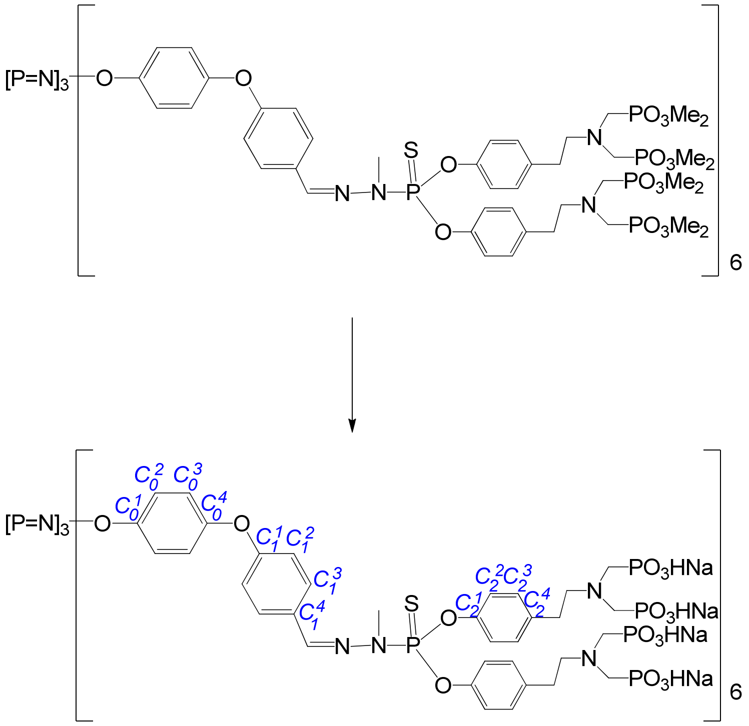

Trimethylsilyl bromide (535 µL, 4.00 mmol) is added to a solution of the aryl ether dendrimer with dimethylphosphonate ends (440 mg, 5.75 10^{-2} mmol) in acetonitrile (10 mL) at 0°C. The mixture is stirred at 25 °C during 12 h then evaporated to dryness under reduced pressure. The crude residue is treated with methanol (2 x 15 mL), washed with ether (20 mL) and suspended in water (1 mL/100 mg) in the presence of one equivalent of NaOH for one phosphonic end. The solution is filtered on microfilters (0.2 µm) and then freeze-dried to afford the dendrimer with sodium salt of phosphonic acid ends (**2-G$_1$**) as a white solid (yield: 85%).

^{31}P-{^{1}H} NMR (D_2O/CD_3CN 7:3, 121.5 MHz): δ = 6.82 (s, PO_3HNa); 10.43 (s, N_3P_3); 13.82 (s, PO_3Na_2); 64.56 (s, P_1). ^{1}H NMR (H_2O/CD_3CN 7:3; 300.13 MHz): δ = 3.25 (AA' part of a AA'BB' system, br s, 24H, <u>CH$_2$</u>-CH$_2$-N); 3.51 (d, $^{2}J_{HP}$ = 11.7 Hz, 48H, N-CH$_2$-P and 18H, CH$_3$-N-P$_1$); 3.82 (BB' part of a AA'BB' system, br s, 24H, CH$_2$-<u>CH$_2$</u>-N); 7.06 (br s, 36 H, C_0^2-H, C_0^3-H, C_1^2-H); 7.29 (br s, 24H, C_2^2-H); 7.51 (br s, 24H, C_2^3-H); 7.78 (br s, 6H, CH=N); 7.96 (br s, 12H, C_1^3-H). ^{13}C-{^{1}H} NMR (D_2O/CD_3CN 7:3, 75.6 MHz): δ = 29.01 (s, <u>CH$_2$</u>-CH$_2$-N); 32.61 (br s, CH$_3$-N-P$_1$); 53.62 (d, $^{1}J_{CP}$ = 128.1 Hz, N-CH$_2$-P); 57.77 (s, CH$_2$-<u>CH$_2$</u>-N); 120.49 (s, C_1^2); 121.57 (s, C_0^3); 121.65 (d, $^{3}J_{CP}$ = 4.5 Hz, C_2^2); 122.57 (s, C_0^2); 128.93 (s, C_1^3); 130.52 (s, C_1^4); 130.85 (s, C_2^3); 134.55 (s, C_2^4); 141.00 (br s, CH=N); 146.01 (br s, C_0^1); 149.39 (d, $^{2}J_{CP}$ = 6.1 Hz, C_2^1); 153.79 (s, C_0^4); 158.17 (s, C_1^1) ppm.

Synthesis and characterization of the monomer for 10

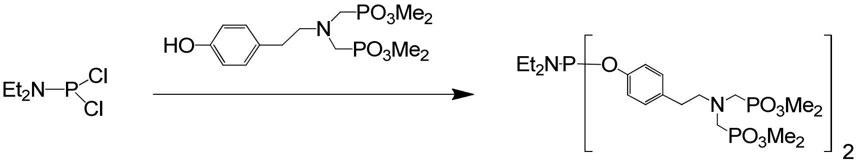

The experimental protocol used for preparing this molecule was inspired by that used by Salamonczyk in order to synthesize thiophosphite dendrimers[2]. The azabisphosphonate tyramine derivative is weighed in a Schlenk tube under argon (2.3 g) and dissolved in 10 mL of distilled THF. The diethylaminodichlorophosphine (0.5 mL) is introduced into another Schlenk tube and 5 mL of distilled THF are added via syringe. Both Schlenk tubes are cooled down to -70°C. 1.4 mL of triethylamine are then added to the dichlorophosphine solution then the tyramine azabisphosphonate solution is added dropwise on the mixture at -70°C via canula. The stirring is continued for half an hour at -70°C then the cooling bath is removed and the stirring is maintained for 4 hours. The mixture is filtered on celite under argon then the solvent is eliminated under reduced pressure. The dry residue **10** is kept under argon at a low temperature and rapidly used without other treatment in the following step.

^{31}P-{^{1}H} NMR ($CDCl_3$, 81.0 MHz): δ = 29.7 (s, PO_3Me_2); 145.0 (s, Et_2NP) ppm. ^{1}H NMR ($CDCl_3$, 250.1 MHz): δ = 1.00 (t, $^{3}J_{HH}$ = 7.2 Hz, 6H, C$\underline{H}_3$CH$_2$); 2.70 (m, 4H, N-C$\underline{H}_2$CH$_2$); 3.00 (m, 4H, CH$_2$C$\underline{H}_2$P); 3.11-3.23 (m, 12H, CH$_2$P, CH$_3$C$\underline{H}_2$); 3.69 (d, $^{3}J_{HP}$ = 6.9 Hz, 24H, CH$_3$O); 6.90 (d, $^{3}J_{HH}$ = 8.4 Hz, 4H, C^{2}H); 6.97 (d, $^{3}J_{HH}$ = 8.4 Hz, 4H, C^{3}H) ppm.

Synthesis and characterization of dendrimer 3-G$_1$

First step

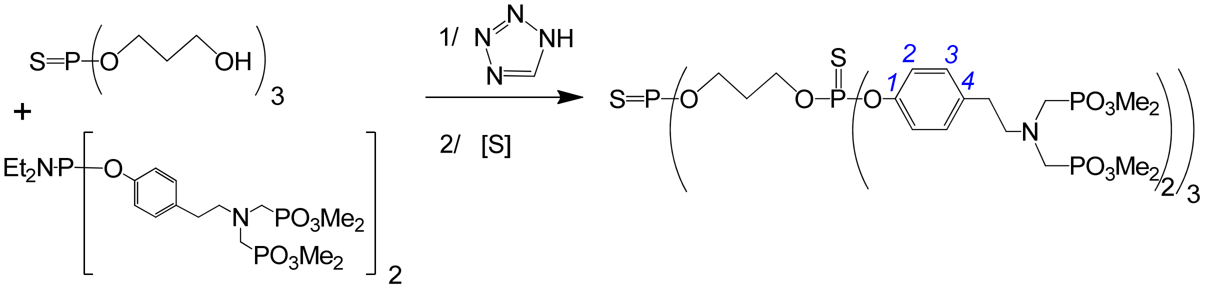

To a solution of the diethylaminophosphine monomer **10** (2.95 g, 3.41 mmol) in dichloromethane (10 mL) is added a solution of (S)P(O(CH$_2$)$_3$OH)$_3$ (219 mg, 0.76 mmol)[2] in dichloromethane (l0 mL) at room temperature. The mixture is then added to a solution of

tetrazole (400 mg, 5.7 mmol) in acetonitrile (5 mL). The resulting solution is stirred for 3 hours at room temperature under argon, then a spatula tip's worth of sulphur is added and stirring is continued at RT for approximately 5 days, until reaction completion (^{31}P NMR control). The mixture is then filtered and the volatiles are removed by evaporation under reduced pressure. The dry residue is subjected to chromatography on silica gel ($CHCl_3$/MeOH, 90:10, Rf = 0.41) to afford the azabisphosphonate terminated dendrimer as a white powder (yield: 72%).

^{31}P-{^{1}H} NMR ($CDCl_3$, 81.0 MHz): δ = 30.3 (s, PO_3Me_2); 62.3 (s, P_1S); 71.7 (s, P_0S) ppm. ^{1}H NMR ($CDCl_3$, 250.1 MHz): δ = 2.08 (q, $^{3}J_{HH}$ = 5.7 Hz, 6H, $CH_2C\underline{H}_2CH_2$); 2.75 (m, 12H, $C\underline{H}_2C_6H_4$); 3.04 (m, 12H, $NC\underline{H}_2$); 3.19 (d, $^{2}J_{HP}$ = 9.2 Hz, 24H, CH_2P); 3.73 (d, $^{3}J_{HP}$ = 10.5 Hz, 72H, CH_3O); 4.18 (dt, $^{3}J_{HH}$ = 5.7 Hz, $^{3}J_{HP}$ = 8.7 Hz, 6H, $CH_2OP(S)$); 4.33 (dt, $^{3}J_{HH}$ = 6.2 Hz, $^{3}J_{HP}$ = 9.2 Hz, 6H, $CH_2OP(S)$); 7.05 (d, $^{3}J_{HH}$ = 7.8 Hz, 12H, C^2H); 7.18 (d, $^{3}J_{HH}$ = 7.8 Hz, 12H, C^3H) ppm. ^{13}C-{^{1}H} NMR ($CDCl_3$, 62.9 MHz): δ = 30.7 (br d, $^{3}J_{CP}$ = 7.6 Hz, $CH_2\underline{C}H_2CH_2$); 33.0 (s, $\underline{C}H_2C_6H_4$); 49.4 (dd, $^{1}J_{CP}$ = 159.0 Hz, $^{3}J_{CP}$ = 7.7 Hz, NCH_2P); 52.8 (d, $^{2}J_{CP}$ = 7.4 Hz, OCH_3); 58.2 (t, $^{3}J_{CP}$ = 7.6 Hz, $CH_2\underline{C}H_2N$); 64.4 (d, $^{2}J_{CP}$ = 3.7 Hz, $P(S)O\underline{C}H_2CH_2CH_2$); 65.5 (d, $^{2}J_{CP}$ = 5.2 Hz, $\underline{C}H_2OP(S)OC_6H_4$); 120.8 (d, $^{3}J_{CP}$ = 5.0 Hz, C^2); 130.0 (s, C^3); 136.6 (s, C^4); 148.9 (d, $^{2}J_{CP}$ = 7.2 Hz, C^1) ppm.

Second step

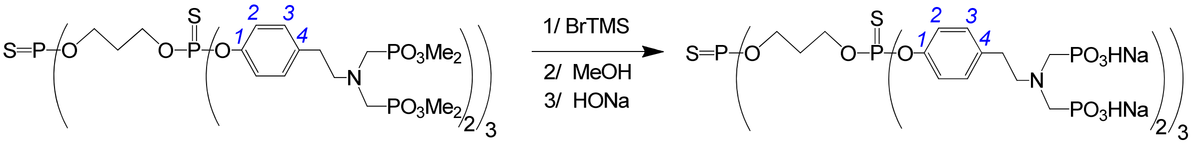

To a solution of dendrimer with azabisphosphonate ends (350 mg, 127 µmol) in solution in dry acetonitrile (5 mL) is added BrTMS (420 µL, 3.18 mmol) dropwise via syringe at 0°C. After 30 minutes at 0°C, the ice bath is removed and stirring is continued overnight at RT. The mixture is evaporated to dryness under reduced pressure. The crude residue is treated twice with methanol (15 mL) for 30 minutes and then washed with water (5 mL) and ether (20 mL) and dried under reduced pressure. The resulting white powder is suspended in water (1 mL/100 mg) in the presence of one equivalent of NaOH for one phosphonic end. The solution is filtered (0.2 µm) and then freeze-dried to afford the dendrimer with sodium salt of phosphonic acid ends **3-G$_1$** as a white solid (yield: 85%).

^{31}P-{^{1}H} NMR (D_2O/CD_3CN, 81.015 MHz): δ = 10.5 (s, PO_3HNa); 62.8 (s, P_1S); 70.8 (s, P_0S) ppm. ^{1}H NMR (D_2O/CD_3CN, 300.13 MHz): δ = 2.19 (br s, 6H, $CH_2C\underline{H_2}CH_2$); 3.17 (br s, $C\underline{H_2}C_6H_4$); 3.59 (d, $^{2}J_{HP}$ = 12.0 Hz, 24H, CH_2P); 3.74 (br s, 12H, $NC\underline{H_2}$); 4.35 (br s, 6H, $CH_2OP(S)$); 4.49 (br s, 6H, $CH_2OP(S)$); 7.21 (br d, $^{3}J_{HH}$ = 7.5 Hz, 12H, C^2H); 7.41 (br d, $^{3}J_{HH}$ = 7.5 Hz, 12H, C^3H) ppm.^{13}C-{^{1}H} NMR (D_2O/CD_3CN, 62.96 MHz): δ = 31.9 (s, $\underline{C}H_2C_6H_4$); 33.0 (br d, $^{3}J_{CP}$ = 9.0 Hz, $CH_2\underline{C}H_2CH_2$); 54.9 (br d, $^{1}J_{CP}$ = 130.9 Hz, NCH_2P); 60.6 (br s, $CH_2\underline{C}H_2N$); 67.6 (br s, $P(S)O\underline{C}H_2CH_2CH_2$); 69.0 (br s, $\underline{C}H_2OP(S)OC_6H_4$); 124.3 (d, $^{3}J_{CP}$ = 3.4 Hz, C^2); 133.6 (s, C^3); 137.2 (s, C^4); 152.2 (d, $^{2}J_{CP}$ = 7.4 Hz, C^1) ppm.

Synthesis and characterization of dendrimer 3-G_2

First step

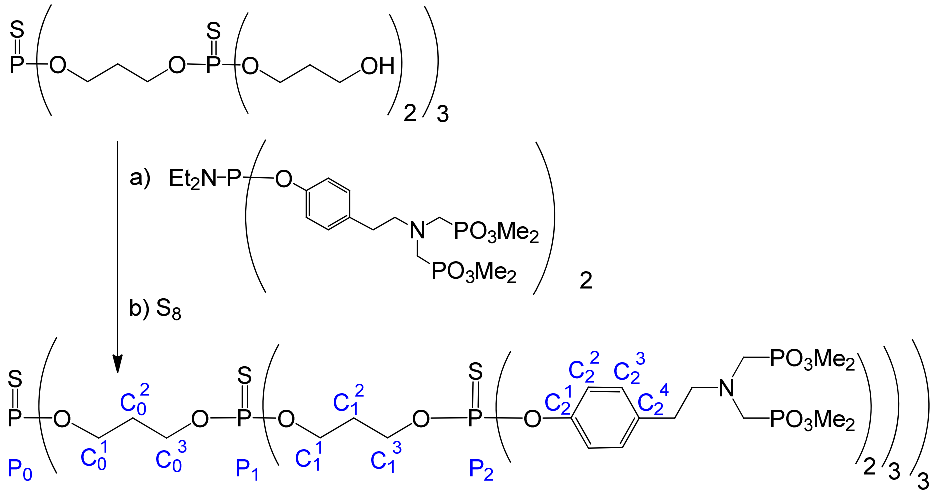

To a solution of the first generation dendrimer Salamonczyk (prepared according to ref[2]) with hydroxyl ends (1.64 g, 1.9 mmol) in solution in dichloromethane (4 mL) are added successively the phosphoramide derived from tyramine aza-bisphosphonate **10** (160 mg, 0.17 mmol) dissolved in dichloromethane/acetonitrile (1 mL/1 mL) and tetrazole (180 mg, 2.60 mmol). The mixture is stirred at 25 °C during 210 min before S_8 (512 mg, 2 mmol) is added. The heterogeneous mixture is stirred during 12 h at 25 °C then filtered. The filtrate is evaporated to dryness under reduced pressure and purified by chromatography on silica gel (dichloromethane/acetone 1:1 to 0:100, then acetone/methanol 1:0 to 0:1, Rf = 0.95 in methanol) to afford the dendrimer with dimethylphosphonate ends as a white solid (yield: 43%).

^{31}P-{^{1}H} NMR (acetone-*d6*, 101.2 MHz): δ = 26.5 (s, PO_3Me_2), 58.7 (s, P^2), 68.2 (s, P^0 and P^1); ^{1}H NMR (acetone-*d6*, 300.1 MHz): δ = 2.10-2.15 (m, 18H, C_1^2-H and C_0^2-H); 2.85 (AA' part of a AA'BB' system, br s, 24H, <u>CH_2</u>-CH_2-N); 3.07 (BB' part of a AA'BB' system, br s, 24H, CH_2-<u>CH_2</u>-N); 3.25 (d, $^2J_{HP}$ = 9.9 Hz, 48H, N-CH_2-P); 3.72 (d, $^3J_{HP}$ = 10.5 Hz, 144H, OMe); 4.24 (m, 24H, C_0^1-H, C_0^3-H and C_1^1-H); 4.44 (m, 12H, C_1^3-H); 7.19 (d, $^3J_{HH}$ = 7.7 Hz, 24H, C_2^2-H); 7.36 (d, $^3J_{HH}$ = 7.8 Hz, 24H, C_2^3-H); ^{13}C-{^{1}H} NMR (acetone-*d6*, 75.5 MHz): δ = 30.6 (br s, C_0^2 and C_1^2); 32.2 (s, <u>CH_2</u>-CH_2-N); 49.1 (dd, $^1J_{CP}$ = 157.6 Hz, $^3J_{CP}$ = 8.2 Hz, N-CH_2-P); 52.1 (d, $^2J_{CP}$ = 5.0 Hz, OMe); 58.1 (t, $^3J_{CP}$ = 7.8 Hz, CH_2-<u>CH_2</u>-N); 64.5 (br s, C_0^1, C_0^3 and C_1^3); 65.90 (d, $^2J_{CP}$ = 6.0 Hz, C_1^1); 120.8 (d, $^3J_{CP}$ = 4.5 Hz, C_2^2); 130.3 (s, C_2^3); 137.5 (s, C_2^4); 149.0 (d, $^2J_{CP}$ = 7.6 Hz, C_2^1) ppm.

Second step

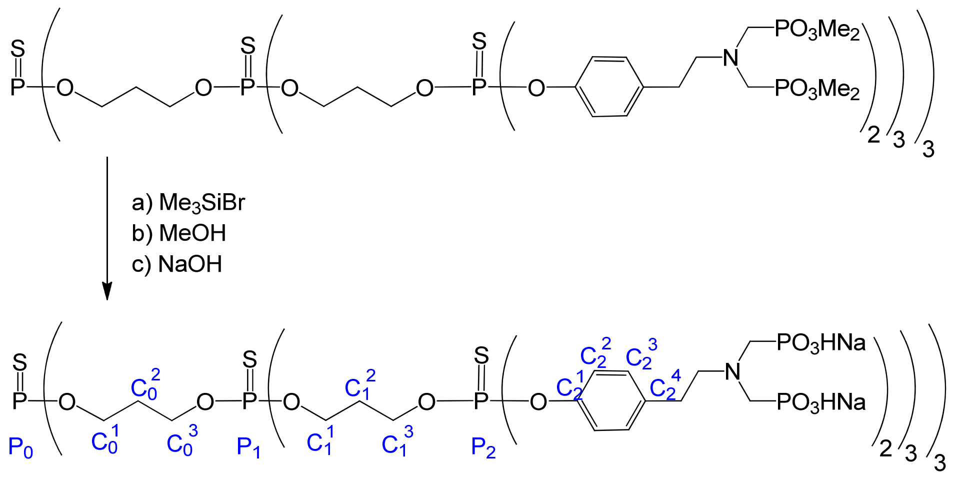

Trimethylsilylbromide (190 µL, 1.44 mmol) is added to a solution of dendrimer with dimethylphosphonate ends (140 mg, 2.39 10^{-2} mmol) in acetonitrile (3.5 mL) at 0°C. The mixture is stirred at 25°C during 12 h then evaporated to dryness under reduced pressure. The residue thus obtained is treated with methanol (2 x 15 mL), washed with ether (20 mL) and suspended in water (1 mL/100 mg) in presence of one equivalent of NaOH for one phosphonic end. The resulting solution was filtered on microfilter (0.2 µm) and then freeze-dried to afford the dendrimer **3-G$_2$** (yield: 70%).

^{31}P-{^{1}H} NMR (D_2O/CD_3CN 9:1, 81.0 MHz): δ = 10.2 (s, PO_3HNa), 62.8 (s, P^2), 70.6 (s, P^0 and P^1); ^{1}H NMR (D_2O/CD_3CN 9:1, 300.1 MHz): δ = 1.97-2.16 (m, 18H, C_1^2-H and C_0^2-H); 3.07 (AA' part of a AA'BB' system, br s, 24H, <u>CH_2</u>-CH_2-N); 3.40 (br s, 48H, N-CH_2-P); 3.63 (BB' part of a AA'BB' system, br s, 24H, CH_2-CH_2-N); 4.13 (br s, 24H, C_0^1-H, C_0^3-H and C_1^1-H); 4.38 (br s, 12H, C_1^3-H); 7.12 (m, 24H, C_2^2-H); 7.31 (m, 24H, C_2^3-H); ^{13}C-{^{1}H} NMR

($CDCl_3$; 75.5 MHz): δ = 29.1 (s, $\underline{CH_2}$-CH_2-N); 32.3 (m, C_0^2 and C_1^2); 52.6 (d, $^1J_{CP}$ = 131.7 Hz, N-CH_2-P); 58.0 (br s, CH_2-$\underline{CH_2}$-N); 64.9 (m, C_0^1, C_0^3, C_1^1 and C_1^3); 121.4 (s, C_2^2); 130.8 (s, C_2^3); 134.5 (s, C_2^4); 149.3 (d, $^2J_{CP}$ = 7.2 Hz, C_2^1).

Synthesis and characterization of dendrimer 4-G_1

Cl/I exchange

The Cl/I exchange in the α position of a silicon atom has been described in the literature in the case of another molecule. Hence, the procedure used here has been adapted from ref[5].

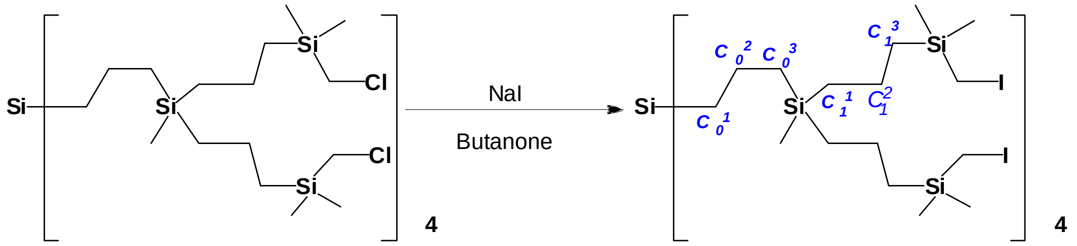

To a solution of the Cl-ended carbosilane dendrimer (crude product, 0.60 mmol) prepared according to a published procedure[3] in dry butanone (8 mL), was added sodium iodide (2.9 g, 19.2 mmol). The reaction mixture was heated to reflux for 12 hours, then allowed to cool to room temperature and filtered. The filtrate was evaporated to dryness under reduced pressure and dissolved in diethyl ether (15 mL). The solution was washed twice with water (2 x 15 mL) and the aqueous layers were extracted with diethyl ether (15 mL). The organic layers were gathered, dried over magnesium sulfate and concentrated under reduced pressure to afford the iodine-terminated carbosilane dendrimer as a pale yellow oil (yield: 85%, purity: ≈ 90%). It should be noted that small amounts of impurities were present as indicated by the ^1H and ^{13}C, but they did not interfere in the subsequent step. The purification was thus delayed and only performed on dendrimer with azabisphosphonate terminal groups.

^1H NMR ($CDCl_3$, 300.1 MHz): δ = -0.03 (s, 12H, Si-C$\underline{H_3}$); 0.15 (s, 48H, Si-(C$\underline{H_3}$)$_2$); 0.55-0.63 (m, 32H, C_0^1-H, C_0^3-H, C_1^1-H); 0.72-0.77 (m, 16H, C_1^3-H); 1.20-1.42 (m, 24H, C_0^2-H, C_1^2-H); 2.02 (s, 16H, Si-C$\underline{H_2}$-I); ^{13}C-{^1H} NMR ($CDCl_3$, 75.6 MHz): δ = -13.0 (s, Si-$\underline{C}H_2$-I); -4.8 (s, Si-$\underline{C}H_3$); -2.8 (s, Si-($\underline{C}H_3$)$_2$); 17.8 (s, C_0^2); 18.4 (s, C_1^2); 18.7 (s, C_0^1, C_1^1); 19.2 (s, C_0^3); 19.6 (s, C_1^3); ^{29}Si NMR ($CDCl_3$, 79.5 MHz): δ = 1.0 (s, $\underline{Si}$-CH_3); 4.0 (s, $\underline{Si}$-CH_2-I, Si core) ppm.

First step

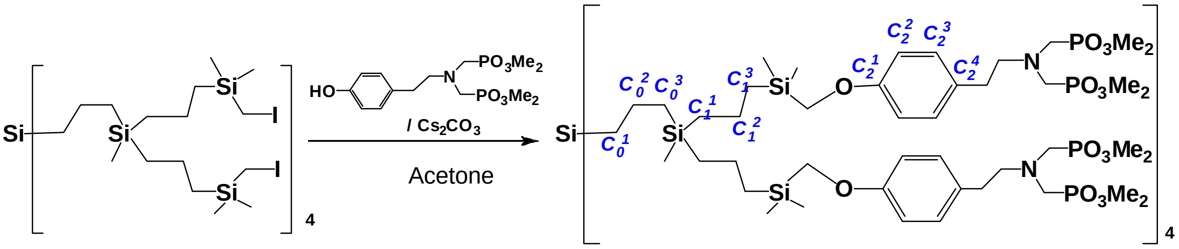

Tyramine aza-bisphosphonate **9** (320 mg, 0.830 mmol) and cesium carbonate (540 mg, 1.66 mmol) are added to a solution of carbosilane type dendrimer of first generation with $SiCH_2I$ ends (200 mg, $8.7.10^{-5}$ mmol) in acetone (2 mL). The mixture is stirred at 40°C during 20 h, centrifuged and the resulting clear solution is evaporated to dryness under reduced pressure. The resulting oil is purified by chromatography on silica gel (acetone/ ethanol, 100:0 to 0:100, Rf = 0.38 in acetone/methanol 9:1) to afford the dendrimer with dimethylphosphonate ends as a pale yellow solid (yield: 65%).

^{31}P-{^{1}H} NMR (acetone-*d6*, 121.5 MHz): δ = 26.5 (s, PO_3Me_2); ^{1}H NMR ($CDCl_3$, 300.1 MHz): δ = 0.00 (s, 12H, Si-CH_3); 0.14 (s, 48H, Si-$(CH_3)_2$); 0.70 (br s, 32H, C_0^1-H, C_0^3-H, C_1^1-H); 0.76-0.81 (m, 16H, C_1^3-H); 1.47-1.52 (m, 24H, C_0^2-H, C_1^2-H); 2.73-2.78 (AA' part of a AA'BB' system, m, 16H, <u>CH_2</u>-CH_2-N); 3.00-3.05 (BB' part of a AA'BB' system, m, 16H, CH_2-<u>CH_2</u>-N); 3.22 (d, $^{2}J_{HP}$ = 9.3 Hz, 32H, N-CH_2-P); 3.61 (s, 16H, Si-CH_2-O); 3.73 (d, $^{3}J_{HP}$ = 10.4 Hz, 96H, OMe); 6.89 (d, $^{3}J_{HH}$ = 8.4 Hz, 16H, C_2^2-H); 7.18 (d, $^{3}J_{HH}$ = 8.4 Hz, 16H, C_2^3-H); ^{13}C-{^{1}H} NMR (CD_3COCD_3, 100.6 MHz): δ = -5.1 (s, Si-CH_3); 17.6 (s, C_0^2, C_1^2); 18.3-18.7 (m, C_1^3, C_1^1, C_0^1, C_0^3); 32.2 (s, <u>CH_2</u>-CH_2-N); 49.1 (dd, $^{1}J_{CP}$ = 157.9 Hz, $^{3}J_{CP}$ = 8.1 Hz, N-CH_2-P); 51.9 (br s, OMe); 58.7 (t, $^{3}J_{CP}$ = 7.0 Hz, CH_2-<u>CH_2</u>-N); 60.0 (s, Si-CH_2-O); 113.9 (s, C_2^2); 129.6 (s, C_2^3); 131.5 (s, C_2^4); 160.0 (s, C_2^1); ^{29}Si NMR ($CDCl_3$, 79.5 MHz): δ = -0.3 (s, Si-CH_2-0); 1.1 (s, Si-CH_3); 4.0 (s, Si at the core) ppm.

Second step

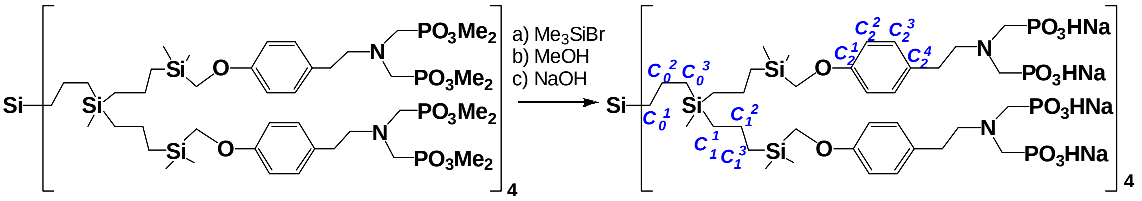

Trimethylsilylbromide (120 µL, $8.56.10^{-1}$ mmol) is added to a solution of dendrimer with dimethylphosphonate endgroups obtained in the previous step (92.9 mg, 2.14 10^{-2} mmol) in acetonitrile (2.5 mL) at 0°C. The mixture is stirred at 25°C during 12 h then evaporated to dryness under reduced pressure. The residue is treated with methanol (2 x 15 mL), washed

with ether (20 mL) and suspended in water (1 mL/100 mg) in the presence of one equivalent of NaOH for one phosphonic end. The solution is filtered on microfilter (0.2 µm) and freeze-dried to afford the dendrimer with sodium salt phosphonic acid ends **4-G_1** as a white solid (yield: 85%).

^{31}P-{^{1}H} NMR (D_2O/acetone-*d6* 7:3, 121.5 MHz): δ = 6.8 (s, PO_3HNa); ^{1}H NMR (D_2O/acetone-*d6* 7:3, 400.13 MHz): δ = -0.14 (br s, 60H, Si-CH_3 and Si-$(CH_3)_2$); 0.45 (br s, 48H, C_0^1-H, C_0^3-H, C_1^1-H, C_1^3-H); 1.23 (br s, 24H, C_0^2-H, C_1^2-H); 2.83 (AA' part of a AA'BB' system, br s, 16H, $\underline{CH_2}$-CH_2-N); 3.11 (br s, 32H, N-CH_2-P); 3.31 (BB' part of a AA'BB' system, br s, 32H, CH_2-$\underline{CH_2}$-N and Si-CH_2-O); 6.65 (m, 16H, C_2^2-H); 7.05 (m, 16H, C_2^3-H); ^{13}C-{^{1}H} NMR (D_2O/acetone-*d6* 7:3, 100.6 MHz): δ = -5.0 (br s, Si-CH_3 and Si-$(CH_3)_2$); 17.5-19.2 (m, C_0^1, C_0^2, C_0^3, C_1^1, C_1^2, C_1^3); 29.3 (s, $\underline{CH_2}$-CH_2-N); 53.7 (d, $^{1}J_{CP}$ = 122.7 Hz, N-CH_2-P); 57.7 (s, CH_2-$\underline{CH_2}$-N); 60.1 (s, Si-CH_2-O, CH_2-$\underline{CH_2}$-N); 114.1 (s, C_2^2); 128.6 (s, C_2^4); 130.1 (s, C_2^3); 159.9 (s, C_2^1) ppm.

Synthesis of carboxylic acid azabis-phosphonate (x = 1) 12a

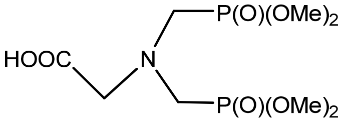

To a solution of aminoacetic acid (5 g, 66.6 mmol) in THF (20 mL) are added 3 equivalents of 37% aqueous formaldehyde solution at room temperature. The solution is stirred for 30 minutes at room temperature and 4 equivalents of dimethylphosphite are then added via syringe. The mixture is maintained under magnetic stirring at room temperature for 12 h. 40 mL of distilled water are then added to the reaction medium, the THF is eliminated under reduced pressure and the product is extracted with 3 x 100 mL of chloroform. The organic phases are dried over magnesium sulfate then evaporated under reduced pressure. The crude residue is purified by column chromatography on silica gel with a CH_2Cl_2/MeOH mixture (95:5) as eluent (Rf = 0.32), and **12a** is isolated in the form of an off-white powder (yield: 37 %).

^{31}P-{^{1}H} NMR ($CDCl_3$, 81.0 MHz) δ = 30.1 ppm. ^{1}H NMR ($CDCl_3$, 200.1 MHz) δ = 3.30 (d, $^{2}J_{HP}$ = 10.1 Hz, 4H, CH_2-P); 3.70 (s, 2H, CH_2-CO); 3.77 (d, $^{3}J_{HP}$ = 10.6 Hz, 12H, O-CH_3), 5.55 (br s, 1H, COOH) ppm. ^{13}C-{^{1}H} NMR ($CDCl_3$, 62.9 MHz) δ = 49.6 (dd, $^{1}J_{CP}$ = 162.1 Hz, $^{3}J_{CP}$ = 9.9 Hz, CH_2-P); 53.1 (d, $^{2}J_{CP}$ = 5.9 Hz, CH_3-O); 56.1 (t, $^{3}J_{CP}$ = 5.8 Hz, N-$\underline{C}H_2$-CO); 171.9 (s, COOH) ppm.

Synthesis of carboxylic acid azabis-phosphonate (x = 3) 12b

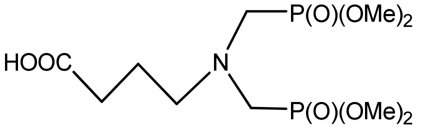

To a solution of aminoacetic acid (5 g, 66.6 mmol) in THF (20mL) are added 3 equivalents of 37% aqueous formaldehyde solution at room temperature. The solution is stirred for 30 minutes at room temperature and 4 equivalents of dimethylphosphite are then added via syringe. The mixture is maintained under magnetic stirring at room temperature for 12 h. 40 mL of distilled water are then added to the reaction medium, the THF is eliminated under reduced pressure and the product is extracted with 3 x 100 mL of chloroform. The organic phases are dried over magnesium sulfate then evaporated under reduced pressure. The crude residue is purified by column chromatography on silica gel with a CH_2Cl_2/MeOH mixture (95:5) as eluent (Rf = 0.35, CH_2Cl_2:MeOH, 95/5), and **12b** is isolated in the form of an off-white powder (yield: 35 %).

^{31}P-{^{1}H} NMR ($CDCl_3$, 81.01 MHz) δ = 30.7 ppm. ^{1}H NMR ($CDCl_3$, 200.1 MHz) δ = 1.74 (quint, $^3J_{HH}$ = 7.1 Hz, 2H, CH_2-C$\underline{H}_2$-CH_2); 2.36 (t, $^3J_{HH}$ = 7.1 Hz, 2H, HOOC-C$\underline{H}_2$); 2.77 (t, $^3J_{HH}$ = 7.1 Hz, 2H, CH_2-C$\underline{H}_2$-N); 3.10 (d, $^2J_{HP}$ = 8.8 Hz, 4H, CH_2-P); 3.74 (d, $^3J_{HP}$ = 10.7 Hz, 12H, O-CH_3); 5.15 (br s, 1H, COOH) ppm. ^{13}C-{^{1}H} NMR ($CDCl_3$, 50.3 MHz) δ = 22.6 (s, CH_2-$\underline{C}H_2$-CH_2); 31.1 (s, HOOC-$\underline{C}H_2$); 49.4 (dd, $^1J_{CP}$ = 158.0 Hz, $^3J_{CP}$ = 7.3 Hz, CH_2-P); 52.8 (d, $^2J_{CP}$ = 7.2 Hz, CH_3-O); 56.0 (t, $^3J_{CP}$ = 7.5 Hz, N-$\underline{C}H_2$- CH_2); 176.1 (s, COOH) ppm.

Synthesis and characterization of amido-tyramine-azabis-phosphonate monomer (x = 1) 11a

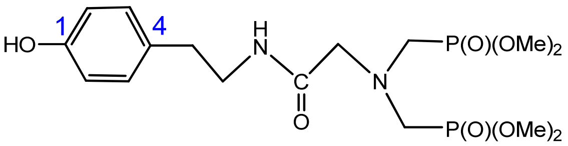

To a solution of carboxylic acid azabisphosphonate (300 mg, 0.94 mmol) (x = 1) in 5 mL of dry DMF at 0°C, 1.3 equivalents of HOBt are added and stirring is continued for 15 minutes at 0°C then 1.3 equivalents of DCC are added. The mixture is stirred for 30 minutes at 0°C then for 1 h at room temperature. The formation of a white precipitate is observed. The mixture is again cooled down to 0°C then tyramine (1.1 equivalents) is added and the reaction mixture is stirred for 30 minutes at 0°C then 15 h at room temperature. The precipitate is eliminated on a 5 µ Millipore filter and the solution is freeze-dried. The residual oil is purified

by column chromatography on silica gel using as eluent a CH_2Cl_2/MeOH mixture (95:5, Rf = 0.15) to afford the expected product **11a** as an off-white powder (yield: 42%).

^{31}P-{^{1}H} NMR ($CDCl_3$, 81.0 MHz) δ = 30.2 ppm. ^{1}H NMR ($CDCl_3$, 200.1 MHz) δ = 2.73 (t, $^{3}J_{HH}$ = 7.4 Hz, 2H, C_6H_4-C$\underline{H}_2$); 3.13 (d, $^{2}J_{HP}$ = 9.0 Hz, 4H, CH_2-P); 3.33-3.52 (m, 4H, CO-CH_2-N, C$\underline{H}_2$-NH); 3.74 (d, $^{3}J_{HP}$ = 10.7 Hz, 12H, CH_3-O); 6.75 (d, $^{3}J_{HH}$ = 8.4 Hz, 2H, C^2H); 7.00 (d, $^{3}J_{HH}$ = 8.4 Hz, 2H, C^3H); 7.46 (t, $^{3}J_{HH}$ = 5.8 Hz, 1H, NH) ppm. ^{13}C-{^{1}H} NMR ($CDCl_3$, 50.3 MHz) δ = 34.7 (s, C_6H_4-$\underline{C}H_2$); 40.7 (s, CH_2-NH); 49.9 (dd, $^{1}J_{CP}$ = 158.9 Hz, $^{3}J_{CP}$ = 3.7 Hz, CH_2-P); 52.8 (d, $^{2}J_{CP}$ = 3.7 Hz, CH_3-O); 60.7 (t, $^{3}J_{CP}$ = 6.4 Hz, CO-$\underline{C}H_2$-N); 115.4 (s, C^2); 129.7 (s, C^3, C^4); 155.4 (s, C^1); 169.7 (s, CONH) ppm.

Synthesis and characterization of amido-tyramine-azabis-phosphonate monomer (x = 3) 11b

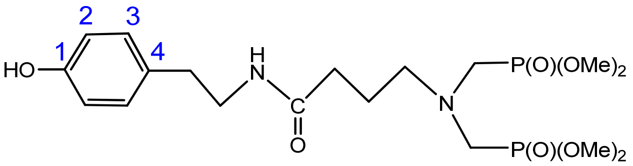

To a solution of carboxylic acid azabisphosphonate (300 mg, 0.86 mmol) (x = 3) in 5 mL of dry DMF at 0°C, are added 1.3 equivalents of HOBt, and stirring is continued for 15 minutes at 0°C then 1.3 equivalents of DCC are added. The mixture is stirred for 30 minutes at 0°C then for 1 h at room temperature. The formation of a white precipitate is observed. The mixture is cooled down to 0°C then tyramine (11 equivalents) is added. The reaction mixture is stirred for 30 minutes at 0°C then 15 h at room temperature. The precipitate is eliminated on a 5 µ Millipore filter and the clear solution is freeze-dried. The residual oil is purified by column chromatography on silica (CH_2C1_2/MeOH, 95:5, Rf = 0.15) to afford the expected compound as a white solid (yield: 51%).

^{31}P-{^{1}H} NMR ($CDCl_3$, 81.01 MHz) δ = 30.6 ppm. ^{1}H NMR ($CDCl_3$, 200.1 MHz) δ = 1.71 (quint, $^{3}J_{HH}$ = 6.8 Hz, 2H, CH_2-C$\underline{H}_2$-CH_2); 2.20 (t, $^{3}J_{HH}$ = 6.8 Hz, 2H, CO-C$\underline{H}_2$-CH_2-CH_2); 2.69 (m, 4H, CO-CH_2-CH_2-C$\underline{H}_2$, C_6H_4-C$\underline{H}_2$); 3.08 (d, $^{2}J_{HP}$ = 8.7 Hz, 4H, CH_2-P); 3.42 (td, $^{3}J_{HH}$ = 7.1 Hz, 2H, C$\underline{H}_2$-NH), 3.75 (d, $^{3}J_{HP}$ = 10.5 Hz, 12H, CH_3-O); 6.67 (t, $^{3}J_{HH}$ = 7.1 Hz, 1H, NH); 6.76 (d, $^{3}J_{HH}$ = 8.4 Hz, 2H, C^2H); 6.98 (d, $^{3}J_{HH}$ = 8.4 Hz, 2H, C^3H); 8.34 (br s, OH) ppm. ^{13}C-{^{1}H} NMR ($CDCl_3$, 62.9 MHz) δ = 23.9 (s, CH_2-$\underline{C}H_2$-CH_2); 33.4 (s, CO-$\underline{C}H_2$-CH_2-CH_2); 34.6 (s, C_6H_4-$\underline{C}H_2$); 40.8 (s, CH_2-NH); 49.5 (dd, $^{1}J_{CP}$ = 159.4 Hz, $^{3}J_{CP}$ = 7.0 Hz, CH_2-P); 52.8 (d, $^{2}J_{CP}$ = 6.2 Hz, CH_3-O), 56.0 (t, $^{3}J_{CP}$ = 7.8 Hz, CO-CH_2-CH_2-$\underline{C}H_2$); 115.5 (s, C^2); 129.7 (s, C^3); 129.7 (s, C^4); 155.4 (s, C^1); 173.6 (s, CONH) ppm.

Synthesis and characterization of dendrimer 5a-G_1

First step

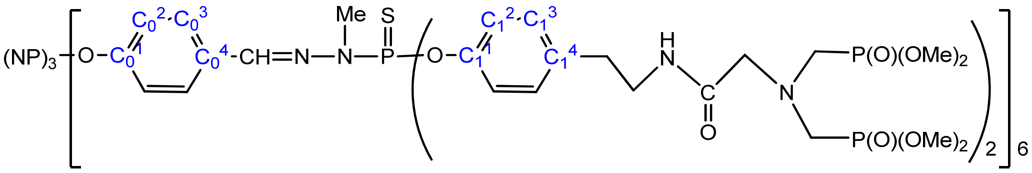

To a solution of generation 1 (12 Cl end groups) phosphorus-containing dendrimer[4] (0.017 mmol) in 3 mL of dry THF are successively added cesium carbonate (5.04 mmol) then tyramine amido-azabis-phosphonate compound **11a** (x = 1) (0.23 mmol) in solution in 3 mL of dry THF. The mixture is stirred overnight at room temperature then filtered on celite. The reaction medium is evaporated under reduced pressure then the dry residue is dissolved in a minimum volume of dichloromethane. The product is then precipitated in a large volume of ether. This operation is repeated three times in order to eliminate the slight excess of starting phenol. The dendrimer is obtained in the form of an off-white powder with (yield: 88%).

^{31}P-{^{1}H} NMR ($CDCl_3$, 81.01 MHz) δ = 11.7 (s, N_3P_3); 30.1 (s, PO_3Me_2); 66.6 (s, P=S) ppm. ^{1}H NMR ($CDCl_3$, 200.1 MHz) δ = 2.77 (br t, $^3J_{HH}$ = 6.8 Hz, 24H, C<u>H</u>$_2$-CH_2-N); 3.12 (d, $^3J_{HP}$ = 9.4 Hz, 48H, P-CH_2); 3.22 (d, $^3J_{HP}$ = 10.1 Hz, 18H, N-CH_3); 3.41-3.48 (m, 48H, C<u>H</u>$_2$-NH, CO-CH_2-N); 3.72 (d, $^2J_{HP}$ = 10.7 Hz, 144H, OMe); 6.97-7.15, 7.50-7.64 (m, 90H, H_{Ar}, CH=N, NH) ppm. ^{13}C-{^{1}H} NMR ($CDCl_3$, 62.90 MHz) δ = 33.0 (d, $^2J_{CP}$ = 12.0 Hz, CH_3-N); 35.0 (s, C_6H_4-<u>C</u>H_2); 40.4 (s, CH_2-NH); 49.9 (dd, $^1J_{CP}$ = 158.4 Hz, $^3J_{CP}$ = 6.0 Hz, CH_2P); 52.8 (s, OMe); 60.8 (br s, CO-<u>C</u>H_2-N); 121.3 (d, $^3J_{CP}$ = 3.7 Hz, C_0^2, C_1^2); 128.3 (s, C_0^3); 129.8 (s, C_1^3); 132.2 (s, C_0^4); 136.2 (s, C_1^4); 138.8 (d, $^3J_{CP}$ = 11.1 Hz, CH=N); 149.9 (d, $^2J_{CP}$ = 6.0 Hz, C_1^1); 151.2 (br s, C_0^1); 169.6 (s, CONH) ppm.

Second step

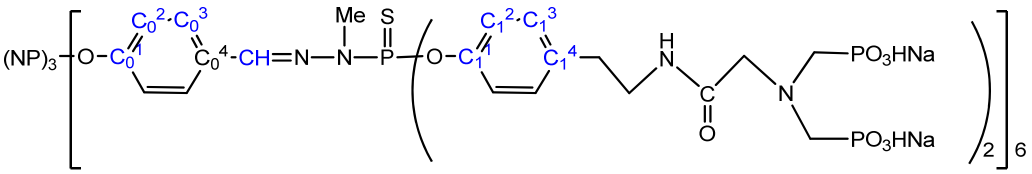

To a solution of dendrimer (0.015 mmol) with amido-azabis-phosphonate ends in solution in 3 mL of distilled acetonitrile at 0°C are added dropwise 48 equivalents of BrTMS (0.73 mmol) under argon. The reaction mixture is stirred for 30 minutes at 0°C then overnight at room temperature. The solution is evaporated to dryness under reduced pressure. The residue

is treated with methanol (2 x 15 mL), washed with ether (20 mL) to afford the phosphonic acid terminated dendrimer (^{31}P-{^{1}H} NMR (D_2O/THFd8, 81.0 MHz) δ = 11.9 (s, PO_3H_2); 12.8 (s, N_3P_3); 66.8 (s, P=S) ppm) and suspended in water (1 mL/100 mg) in the presence of one equivalent of NaOH for one phosphonic end. The solution is filtered on microfilter (0.2 µm) and freeze-dried to afford the dendrimer with sodium salt phosphonic acid ends **5a-G$_1$** as a white solid (yield: 63%).

^{31}P-{^{1}H} NMR (D_2O/THFd8, 81.0 MHz) δ = 9.5 (s, PO_3HNa); 12.9 (s, N_3P_3); 66.8 (s, P=S) ppm. ^{1}H NMR (D_2O/THFd8, 200.1 MHz) δ = 1.25 (br s, 24H, C_6H_4-CH_2); 2.78 (br s, ??); 3.09 (d, $^{3}J_{PH}$ = 10.6 Hz, 48H, CH_2P); 3.07-3.35 (m, 42H, NCH_3, *CH_2*NH); 3.86 (br s, 24H, $COCH_2$); 6.80 (d, $^{3}J_{HH}$ = 8.5 Hz, 12H, C_0^2H); 7.02 (d, $^{3}J_{HH}$ = 8.0 Hz, 24H, C_1^2H); 7.21 (d, $^{3}J_{HH}$ = 8.7 Hz, 24H, C_1^3H); 7.65 (d, $^{3}J_{HH}$ = 7.7 Hz, 12H, C_0^3H); 7.89(br s, 6H, CH=N) ppm. ^{13}C-{^{1}H} NMR (D_2O/THFd8, 62.9 MHz) δ = 33.1 (d, $^{3}J_{CP}$ = 12.1 Hz, CH_3-N); 37.0 (s, C_6H_4-$\underline{C}H_2$); 43.6 (s, CH_2-NH); 56.6 (d, $^{1}J_{CP}$ = 140.7 Hz, NCH_2P); 62.0 (br s, CO-$\underline{C}H_2$-N); 121.7 (s, C_0^2, C_1^2); 123.6 (s, C_1^3); 130.9 (s, C_0^3); 132.8 (s, C_1^4); 136.5 (s, C_0^4); 139.6 (br s, CH=N); 151.5 (br s, C_1^1); 153.4 (br s, C_0^1); 172.8 (s, CONH) ppm.

Synthesis and characterization of dendrimer 5b-G$_1$

First step

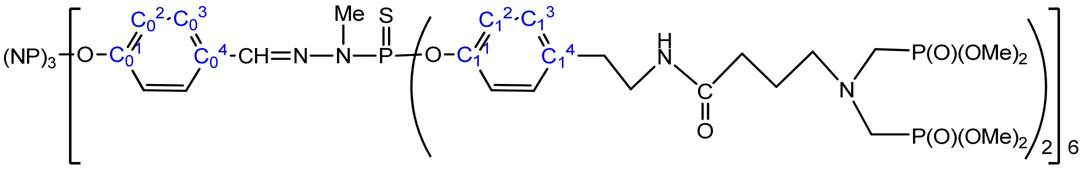

To a solution of generation 1 (12 Cl ends) phosphorus-containing dendrimer[4] (0.017 mmol) in 3 mL of dry THF are successively added cesium carbonate (5.04 mmol) then a solution of tyramine-amido-azabis-phosphonate compound **11b** (x = 3) (0.23 mmol) in 3 mL of dry THF. The mixture is stirred overnight at room temperature then filtered on celite. The reaction medium is evaporated under reduced pressure then the crude residue is dissolved in a minimum volume of dichloromethane. The product is then precipitated in a large volume of ether. This operation is repeated three times in order to eliminate the slight excess of starting phenol. The product is obtained in the form of an off-white powder (yield: 85%).

^{31}P-{^{1}H} NMR ($CDCl_3$, 81.0 MHz): δ = 11.9 (s, N_3P_3); 30.5 (s, PO_3Me_2); 66.5 (s, P=S) ppm. ^{1}H NMR ($CDCl_3$, 200.0 MHz): δ = 1.67 (quint, $^{3}J_{HH}$ = 6.2 Hz, 24H, CH_2-C$\underline{H}_2$-CH_2); 2.19 (t, $^{3}J_{HH}$ = 6.2 Hz, 24H, CO-C$\underline{H}_2$-CH_2-CH_2); 2.67-2.70 (m, 48H, CO-CH_2-CH_2-C$\underline{H}_2$, C_6H_4-C$\underline{H}_2$);

3.04 (d, $^{2}J_{HP}$ = 8.9 Hz, 48H, P-CH_2); 3.18 (d, $^{3}J_{HP}$ = 10.2 Hz, 18H, N-CH_3); 3.33-3.42 (m, 24H, C$\underline{H_2}$-NH); 3.70 (d, $^{3}J_{HP}$ = 10.5 Hz, 144H, CH_3-O); 6.91-7.04 (m, 72H, NHCO, H_{Ar}); 7.56-7.61 (m, 18H, H_{Ar}, CH=N) ppm. ^{13}C-{^{1}H} NMR ($CDCl_3$, 62.9 MHz): δ = 24.1 (s, CH_2-$\underline{C}H_2$-CH_2); 33.0 (d, $^{2}J_{CP}$ = 11.8 Hz, CH_3-N); 33.4 (s, CO-$\underline{C}H_2$); 34.9 (s, C_6H_4-$\underline{C}H_2$); 40.5 (s, CH_2-NH); 49.5 (dd, $^{1}J_{CP}$ = 159.3 Hz, $^{3}J_{CP}$ = 6.9 Hz, CH_2P); 52.7 (d, $^{2}J_{CP}$ = 4.9 Hz, OCH_3); 56.1 (t, $^{3}J_{CP}$ = 6.9 Hz, CO-$\underline{C}H_2$-N); 121.1 (s, C_0^2); 121.2 (s, C_1^2); 128.3 (s, C_0^3); 129.8 (s, C_1^3); 132.2 (s, C_0^4); 136.5 (s, C_1^4); 138.7 (d, $^{3}J_{CP}$ = 13.9 Hz, CH=N); 148.9 (d, $^{2}J_{CP}$ = 6.9 Hz, C_1^1); 151.2 (br s, C_0^1); 173.4 (s, CONH) ppm.

Second step

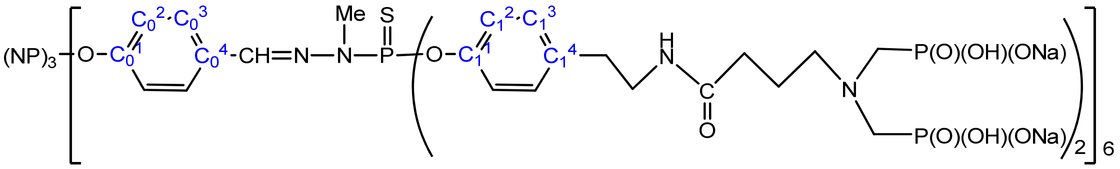

To a solution of dendrimer with amido-azabis-phosphonate ends (0.015 mmol) prepared as described above in 3 mL of distilled acetonitrile at 0°C are added dropwise 48 equivalents of BrTMS (0.73 mmol) under argon. The mixture is stirred for 30 minutes at 0°C then overnight at room temperature. The solution is evaporated to dryness under reduced pressure. The residue is treated with methanol (2 x 15 mL), washed with ether (20 mL) to afford the phosphonic acid terminated dendrimer and suspended in water (1 mL/100 mg) in the presence of one equivalent of NaOH for one phosphonic end. The solution is filtered on microfilter (0.2 µm) and freeze-dried to afford the dendrimer with sodium salt phosphonic acid ends **5b-G$_1$** as a white solid (yield: 72%).

^{31}P-{^{1}H} NMR (D_2O/CD_3COCD_3, 121.5 MHz): δ = 6.7 (s, PO_3HNa); 9.7 (s, N_3P_3); 64.2 (s, P=S) ppm. ^{1}H NMR (D_2O/CD_3COCD_3, 200.1 MHz): δ = 1.97 (br s, 24H, CH_2); 2.20 (br s, 24H, CH_2); 2.58 (br s, 24H, CH_2); 3.02-3.21 (m, 72H, CH_2, CH_2P); 3.44 (br s, 42H, CH_2, NMe); 6.71 (br s, 12H, C_0^2H); 6.95-7.08 (m, 60H, NHCO, C_1^2H, C_1^3H); 7.44 (br s, 12H, C_0^3H), 7.66 (br s, 6H, CH=N) ppm. ^{13}C-{^{1}H} NMR (D_2O/THFd8, 50.3 MHz) δ = 22.8 (s, CH_2-$\underline{C}H_2$-CH_2); 35.0 (br s, CH_3-N, CO-$\underline{C}H_2$); 36.8 (s, C_6H_4-$\underline{C}H_2$); 43.4 (s, CH_2-NH); 53.4 (s, CO-$\underline{C}H_2$-N); 57.4 (d, $^{1}J_{CP}$ = 146.6 Hz, CH_2P); 121.9 (br s, C_0^2, C_1^2); 123.7 (s, C_1^3); 131.0 (s, C_0^3); 132.6 (s, C_1^4); 136.1 (s, C_0^4); 139.3 (s, CH=N); 151.5 (br s, C_1^1); 153.6 (s, C_0^1); 176.6 (s, CONH) ppm.

Synthesis and characterization of dendrimer 6a-G_1

First step

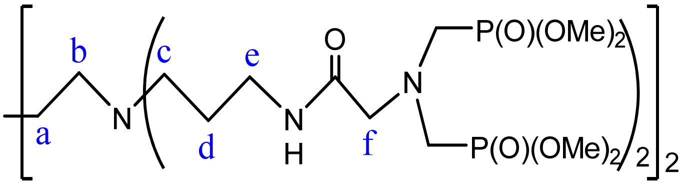

To a solution of aza-bisphosphonate carboxylic acid **12a** (x = 1; 2 mmol) in 4 mL of dry DMF at 0°C is added under an inert atmosphere HOBt (2.6 mmol) and stirring is continued at 0°C for 30 minutes, then DCC (2.6 mmol) is added. After 30 minutes at 0°C the reaction mixture is allowed to reach room temperature and stirring is continued for an additional 1 hour, while the progressive formation of a precipitate is observed. The suspension is cooled down to 0°C then first-generation DAB dendrimer (0.33 mmol) is added. After 30 minutes at 0°C stirring is continued at room temperature for 20 h. The precipitate is eliminated on 5 µm Millipore filters and the resulting clear solution is freeze-dried. The crude residue is treated three times by dissolution in a minimum volume of dichloromethane and precipitation in a large volume of diethylether so as to eliminate excess of reagents. The dendrimer is obtained in the form of an off-white powder (yield: 73%) after purification by column chromatography on silica gel. ^{31}P-{^{1}H} NMR ($CDCl_3$, 81.1 MHz): δ = 30.2 ppm. ^{1}H NMR ($CDCl_3$, 200.0 MHz) δ = 1.68 (br s, 12H, Ha, Hd); 2.49 (br s, 12H, Hb, Hc); 3.20 (d, $^2J_{HP}$ = 9.2 Hz, 16H, CH_2P); 3.27 (s, 8H, He); 3.45 (br s, 8H, Hf); 3.75 (d, $^3J_{HP}$ = 10.6 Hz, 48H, OMe); 7.98 (br s, 4H, CONH) ppm. ^{13}C-{^{1}H} NMR ($CDCl_3$, 50.3 MHz) δ = 24.1 (s, Ca); 26.5 (s, Cd); 37.4 (s, Ce); 49.7 (dd, $^1J_{CP}$ = 157.6 Hz, $^3J_{CP}$ = 6.3 Hz, CH_2P); 51.4 (s, Cb, Cc); 52.8 (d, $^2J_{CP}$ = 5.9 Hz, OMe); 60.5 (t, $^3J_{CP}$ = 6.5 Hz, Cf); 169.7 (s, CONH) ppm.

Second step

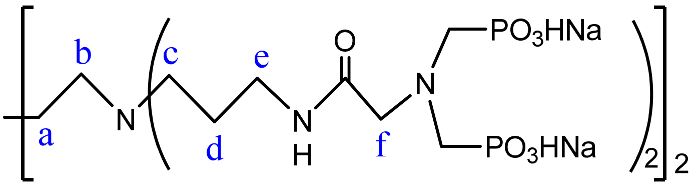

To a solution of generation 1 DAB-type dendrimer with azabis-phosphonate ends as prepared above in 4 mL of freshly distilled acetonitrile at 0°C is added dropwise BrTMS (5.0 mmol). The mixture is stirred at 0°C for 30 minutes then at room temperature for another 15 hours. The solution is evaporated to dryness under reduced pressure. The residue is treated with

methanol (2 x 15 mL), washed with ether (20 mL) and finally twice with a THF/ diethylether mixture (1:9) to afford the phosphonic acid terminated dendrimer (^{31}P-{^{1}H} NMR (D_2O/THFd8, 81.0 MHz): $\delta = 11.5$ ppm (s, PO_3H_2)). The residue is suspended in water (1 mL/100 mg) in the presence of one equivalent of NaOH for one phosphonic end. The solution is filtered on microfilter (0.2 µm) and freeze-dried to afford the dendrimer with sodium salt phosphonic acid ends **6a-G$_1$** as a white solid (yield: 68%).

^{31}P-{^{1}H} NMR (D_2O/THF*d8*, 101.3 MHz): $\delta = 10.0$ ppm. ^{1}H NMR (D_2O/THF*d8*, 250.1 MHz): $\delta = 1.37$ (br s, 4H, Ha); 1.92 (br s, 8H, Ha); 2.08 (br s, 12H, Hd); 3.10 (d, $^{2}J_{HP} = 10.6$ Hz, 16H, CH_2P); 3.33 (s, 8H, He); 3.45 (br s, 8H, Hb, Hc); 3.72 (s, 8H, Hf); 7.49-8.02 (m, NH) ppm. ^{13}C-{^{1}H} NMR (D_2O/CD_3COCD_3, 62.9 MHz): $\delta = 23.7$ (s, Ca); 26.0 (s, Cd); 38.8 (s, Ce); 54.4 (d, $^{1}J_{CP} = 159.6$ Hz, CH_2P); 54.6 (s, Cc); 58.1 (s, Cb); 63.1 (br s, Cf); 175.8 (s, CONH) ppm.

Synthesis and characterization of dendrimer 6b-G$_1$

First step

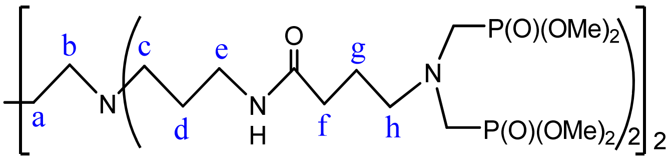

To a solution of aza-bisphosphonate carboxylic acid **12b** (x = 3; 2 mmol) in 4 mL of dry DMF at 0°C is added HOBt (2.6 mmol). The mixture is stirred at 0°C for 30 minutes, and DCC (2.6 mmol) is added. After 30 minutes at 0°C the mixture is allowed to warm to room temperature and stirring is continued for another hour, the formation of a precipitate is observed. The suspension is cooled to 0°C then first-generation DAB dendrimer (0.33 mmol) is added. After 30 minutes at 0°C stirring is continued at room temperature for 20 h. The precipitate is eliminated on 5 µ Millipore filters and the clear solution is freeze-dried. The product is treated three times by dissolution in a minimum volume of dichloromethane and precipitation in a large volume of diethylether so as to eliminate the excess of reagents. The dendrimer is obtained in the form of an off-white powder after purification by column chromatography on silica gel (yield: 69%).

^{31}P-{^{1}H} NMR ($CDCl_3$, 81.0 MHz): $\delta = 30.4$ ppm. ^{1}H NMR ($CDCl_3$, 200.1 MHz): $\delta = 1.70$-1.83 (m, 20H, Ha, Hd, Hg); 2.23 (t, $^{3}J_{HH} = 6.9$ Hz, 8H, Hf); 2.72 (m, 8H, Hh); 2.97 (m, 12H, Hb, Hc); 3.09 (d, $^{3}J_{HH} = 6.9$ Hz, 16H, CH_2P); 3.24 (m, 8H, He); 3.72 (d, $^{3}J_{HP} = 10.5$ Hz, 48H,

OMe); 7.83 (m, 4H, CONH) ppm. ^{13}C-{^{1}H} NMR ($CDCl_3$, 62.9 MHz): δ = 21.5 (s, Ca); 23.6 (s, Cg); 23.9 (s, Cd); 33.2 (s, Cf); 36.4 (s, Ce); 49.4 (dd, $^1J_{CP}$ = 157.8 Hz, $^3J_{CP}$ = 6.5 Hz, CH_2P); 50.5 (s, Cc); 52.2 (s, Cb); 52.7 (d, $^2J_{CP}$ = 5.5 Hz, OMe); 56.2 (t, $^3J_{CP}$ = 6.5 Hz, Ch); 173.9 (s, CONH) ppm.

Second step

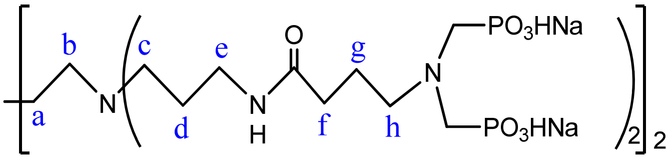

To a solution of generation 1 DAB-type dendrimer with azabis-phosphonate ends as prepared above in 4 mL of freshly distilled acetonitrile at 0°C is added dropwise BrTMS (5.0 mmol). The mixture is stirred at 0°C for 30 minutes then at room temperature for another 15 hours. The solution is evaporated to dryness under reduced pressure. The residue is treated with methanol (2 x 15 mL), washed with ether (20 mL) and finally twice with a THF/ diethylether mixture (1:9) to afford the phosphonic acid terminated dendrimer (^{31}P-{^{1}H} NMR (D_2O/CD_3COCD_3, 81.0 MHz): δ= 11.0 ppm (s, PO_3H_2). The residue is suspended in water (1 mL/100 mg) in the presence of one equivalent of NaOH for one phosphonic end. The solution is filtered on microfilter (0.2 µm) and freeze-dried to afford the dendrimer with sodium salt phosphonic acid ends **6b-G$_1$** as a white solid (yield: 62%).

^{31}P-{^{1}H} NMR (D_2O/CD_3COCD_3, 81.0 MHz): δ = 10.2 (PO_3HNa, major), 17.8 (PO_3Na_2, minor) ppm. ^{1}H NMR (D_2O/THF*d8*, 200.1 MHz): δ = 1.95-2.10 (m, 20H, Ha, Hd, Hh); 2.43 (br s, 8H, Hf); 3.21-3.32 (m, 24H, He, CH_2P); 3.55 (br s, 12H, Hb, Hc); 3.62 (br s, 8H, Hh); 7.57-7.89 (m, 4H, NH) ppm. ^{13}C-{^{1}H} NMR (D_2O/CD_3COCD_3, 62.9 MHz): δ = 22.7 (s, Cg); 23.3 (s, Ca); 26.0 (s, Cd); 35.2 (s, Cf); 39.0 (s, Ce); 52.9 (s, Cc); 54.4 (s, Cb); 55.0 (d, $^1J_{CP}$ = 130.7 Hz, CH_2P); 58.8 (s, Ch); 177.6 (s, CONH) ppm.

Synthesis and characterization of dendrimer 6b-G$_2$

First step

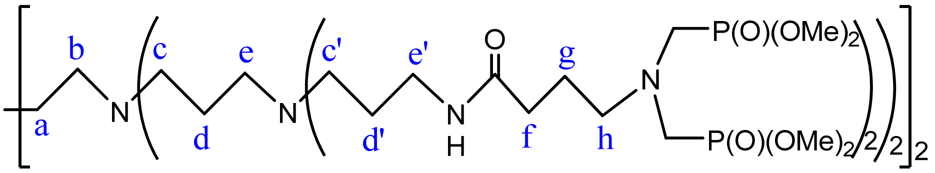

To a solution of aza-bisphosphonate carboxylic acid **12b** (x = 3; 2 mmol) in 4 mL of dry DMF at 0°C is added HOBt (2.6 mmol). The mixture is stirred at 0°C for 30 minutes, and DCC (2.6 mmol) is added. After 30 minutes at 0°C the mixture is allowed to warm to room temperature and stirring is continued for another hour, the formation of a precipitate is observed. The suspension is cooled to 0°C then second-generation DAB dendrimer (0.17 mmol) is added. After 30 minutes at 0°C stirring is continued at room temperature for 20 h. The precipitate is eliminated on 5 μ Millipore filters and the clear solution is freeze-dried. The product is treated three times by dissolution in a minimum volume of dichloromethane and precipitation in a large volume of diethylether so as to eliminate the excess of reagents. The dendrimer is obtained in the form of an off-white powder after purification by column chromatography on silica gel (yield: 75%).

^{31}P-{^{1}H} NMR ($CDCl_3$, 80.0 MHz): $\delta = 30.5$ ppm.

^{1}H NMR ($CDCl_3$, 200.1 MHz): $\delta = 1.62$-1.91 (m, 44H, Ha, Hd, Hd', Hg); 2.19 (br s, 16H, Hf); 2.70 (br s, 16H, Hh); 2.92-3.20 (m, 84H, Hb, Hc, Hc', CH_2P, He, He'); 7.80 (m, 8H, CONH) ppm. ^{13}C-{^{1}H} NMR ($CDCl_3$, 62.9 MHz): $\delta = 20.8$ (s, Ca); 23.6 (s, Cg); 24.4 (s, Cd, Cd'); 33.2 (s, Cf); 36.6 (s, Ce'); 49.3 (dd, $^{1}J_{CP} = 158.1$ Hz, $^{3}J_{CP} = 6.8$ Hz, CH_2P); 50.5 (s, Cc, Cc'); 52.4 (s, Cb, Ce); 52.7 (d, $^{2}J_{CP} = 5.8$ Hz, OMe); 56.2 (t, $^{3}J_{CP} = 6.5$ Hz, Ch); 173.7 (s, CONH) ppm.

Second step

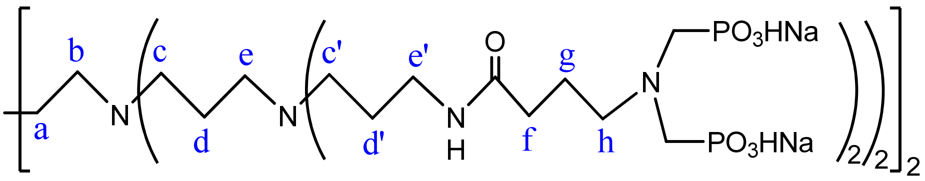

To a solution of generation 2 DAB-type dendrimer with azabis-phosphonate ends as prepared above in 4 mL of freshly distilled acetonitrile at 0°C is added dropwise BrTMS (7.6 mmol). The mixture is stirred at 0°C for 30 minutes then at room temperature for another 15 hours. The solution is evaporated to dryness under reduced pressure. The residue is treated with methanol (2 x 15 mL), washed with ether (20 mL) and finally twice with a THF/ diethylether mixture (1:9) to afford the phosphonic acid terminated dendrimer (^{31}P-{^{1}H} NMR (D_2O/CD_3COCD_3, 81.0 MHz): $\delta = 10.9$ ppm (s, PO_3H_2)). The residue is suspended in water (1 mL/100 mg) in the presence of one equivalent of NaOH for one phosphonic end. The solution is filtered on microfilter (0.2 μm) and freeze-dried to afford the dendrimer with sodium salt phosphonic acid ends **6b-G$_2$** as a white solid (yield: 72%).

$^{31}P-\{^1H\}$ NMR (D_2O/CD_3COCD_3, 81.0 MHz): δ = 10.1 ppm. $^1H\}$ NMR (D_2O/ CD_3COCD_3, 200.1 MHz): δ = 1.90-2.16 (m, 36H, Ha, Hd, Hd', Hg); 2.45 (br s, 16H, Hf); 3.10-3.40 (m, 92H, CH_2P, He'); 3.57 (br s, 36H, Hb, Hc, He, Hc'); 3.63 (br s, 16H, Hh); 7.59-7.90 (m, NH) ppm. $^{13}C-\{^1H\}$ NMR (D_2O/CD_3COCD_3, 62.9 MHz): δ = 21.7 (s, Ca); 22.8 (s, Cg); 25.8 (s, Cd, Cd'); 35.2 (s, Cf); 39.1 (s, Ce'); 52.4 (s, Cc, Cc'); 52.9 (s, Cb, Ce); 55.2 (d, $^1J_{CP}$ = 129.4 Hz, CH_2P); 58.8 (s, Ch); 177.7 (s, CONH) ppm.

Synthesis and characterization of dendrimer 7a-G1

First step

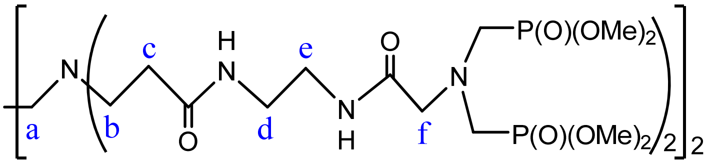

To a solution of aza-bisphosphonate carboxylic acid **12a** (x = 1, 2 mmol) in 4 mL of dry DMF at 0°C is added HOBt (2.6 mmol). The mixture is stirred at 0°C for 30 minutes, and DCC (2.6 mmol) is added. After 30 minutes at 0°C the mixture is allowed to warm to room temperature and stirring is continued for another hour, the formation of a precipitate is observed. The suspension is cooled to 0°C then first-generation PAMAM dendrimer (0.33 mmol) is added. After 30 minutes at 0°C stirring is continued at room temperature for 20 h. The precipitate is eliminated on 5 µ Millipore filters and the clear solution is freeze-dried. The product is treated three times by dissolution in a minimum volume of dichloromethane and precipitation in a large volume of diethylether so as to eliminate the excess of reagents. The dendrimer is obtained in the form of an off-white powder with (yield: 67%) after purification by column chromatography on silica gel.

$^{31}P-\{^1H\}$ NMR ($CDCl_3$, 81.0 MHz): δ = 30.1 ppm. ^1H NMR ($CDCl_3$, 200.1 MHz): δ = 2.48 (br s, 12H, Ha, Hc); 2.98 (m, 8H, Hb); 3.18 (d, $^2J_{HP}$ = 9.4 Hz, 16H, CH_2P); 3.29 (br s, 16H, Hd, He); 3.41 (br s, 8H, Hf); 3.72 (d, $^3J_{HP}$ = 10.6 Hz, 48H, OMe); 7.77 (br s, 4H, CONH); 8.17 (br s, 4H, CONH) ppm. $^{13}C-\{^1H\}$ NMR ($CDCl_3$, 62.9 MHz): δ = 31.6 (s, Cc); 38.9 (s, Ce); 39.2 (s, Cd); 48.8 (s, Cb); 49.7 (s, Ca); 50.2 (dd, $^1J_{CP}$ = 159.2 Hz, $^3J_{CP}$ = 6.5 Hz, CH_2P); 52.8 (d, $^2J_{CP}$ = 5.0 Hz, OMe); 60.7 (t, $^3J_{CP}$ = 6.6 Hz, Cf); 170.3 (s, CONH); 171.4 (s, CONH) ppm.

Second step

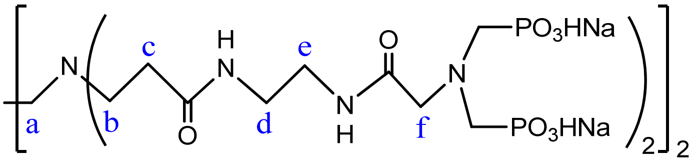

To a solution of generation 1 PAMAM-type dendrimer with azabis-phosphonate ends (0.2 mmol) as prepared above in 4 mL of freshly distilled acetonitrile at 0°C is added dropwise BrTMS (5 mmol). The mixture is stirred at 0°C for 30 minutes then at room temperature for another 15 hours. The solution is evaporated to dryness under reduced pressure. The residue is treated with methanol (2 x 15 mL), washed with ether (20 mL) and finally twice with a THF/ diethylether mixture (1:9) to afford the phosphonic acid terminated dendrimer ($^{31}P-\{^{1}H\}$ NMR (D_2O/THFd8, 81.0 MHz): δ = 10.9 (s, PO_3H_2) ppm). The residue is suspended in water (1 mL/100 mg) in the presence of one equivalent of NaOH for one phosphonic end. The solution is filtered on microfilter (0.2 µm) and freeze-dried to afford the dendrimer with sodium salt phosphonic acid ends **7a-G$_1$** as a white solid (yield: 65%).

$^{31}P-\{^{1}H\}$ NMR (D_2O/CD_3COCD_3, 81.0 MHz): δ = 10.2 (PO_3HNa, major), 19.6 (PO_3Na_2, minor) ppm. ^{1}H NMR (D_2O/CD_3COCD_3, 200.1 MHz): δ = 2.71 (br s, 8H, Hc); 3.07 (d, $^{2}J_{HP}$ = 11.1 Hz, 24H, CH_2P, Hb); 3.26 (br s, 12H, Ha, Hd), 3.40 (br s, 8H, He); 3.69 (br s, 8H, Hf); 7.50-8.80 (m, 8H, NH) ppm.

Synthesis and characterization of dendrimer 7b-G$_1$

First step

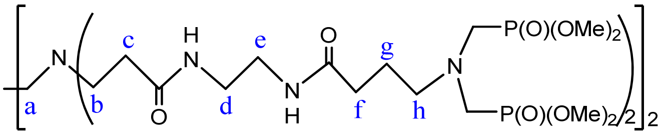

To a solution of aza-bisphosphonate carboxylic acid **12b** (x = 3; 2 mmol) in 4 mL of dry DMF at 0°C is added HOBt (2.6 mmol). The mixture is stirred at 0°C for 30 minutes, and DCC (2.6 mmol) is added. After 30 minutes at 0°C the mixture is allowed to warm to room temperature and stirring is continued for another hour, the formation of a precipitate is observed. The suspension is cooled to 0°C then first-generation PAMAM dendrimer (0.33 mmol) is added. After 30 minutes at 0°C stirring is continued at room temperature for 20 h. The precipitate is eliminated on 5 µ Millipore filters and the clear solution is freeze-dried. The product is treated three times by dissolution in a minimum volume of dichloromethane and precipitation in a

large volume of diethylether so as to eliminate the excess of reagents. The dendrimer is obtained as an off-white powder (yield: 61%) after purification by column chromatography on silica gel.

^{31}P-{^{1}H} NMR ($CDCl_3$, 81.0 MHz): δ = 30.4 ppm. ^{1}H NMR ($CDCl_3$, 200.0 MHz): δ = 1.72 (m, 8H, Hg); 2.21 (t, $^{3}J_{HH}$ = 6.3 Hz, 8H, Hf); 2.55 (m, 8H, Hc); 2.73 (t, $^{3}J_{HH}$ = 6.3 Hz, 8H, Hh); 2.93 (m, 12H, Ha, Hb); 3.09 (d, $^{2}J_{HP}$ = 9.1 Hz, 16H, CH_2P); 3.28 (br s, 16H, Hd, He); 3.73 (d, $^{3}J_{HP}$ = 10.6 Hz, 48H, OMe); 7.65 (br s, 4H, CONH); 8.21 (br s, 4H, CONH) ppm. ^{13}C-{^{1}H} NMR ($CDCl_3$, 62.9 MHz): δ = 23.5 (s, Cg); 32.1 (s, Cc); 33.3 (s, Cf); 39.2 (s, Ce); 39.6 (s, Cd); 49.2 (s, Cb); 49.5 (dd, $^{1}J_{CP}$ = 157.9 Hz, $^{3}J_{CP}$ = 7.2 Hz, CH_2P); 50.1 (s, Ca); 52.6 (d, $^{2}J_{CP}$ = 4.6 Hz, OMe); 56.1 (t, $^{3}J_{CP}$ = 6.7 Hz, Ch); 171.7 (s, CONH); 173.7 (s, CONH) ppm.

Second step

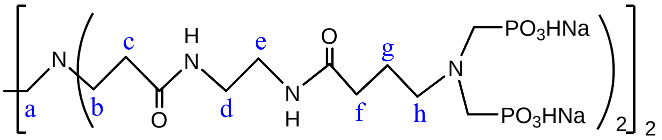

To a solution of generation 1 PAMAM-type dendrimer with azabis-phosphonate ends (0.2 mmol) as prepared above in 4 mL of freshly distilled acetonitrile at 0°C is added dropwise BrTMS (5 mmol). The mixture is stirred at 0°C for 30 minutes then at room temperature for another 15 hours. The solution is evaporated to dryness under reduced pressure. The residue is treated with methanol (2 x 15 mL), washed with ether (20 mL) and finally twice with a THF/ diethylether mixture (1:9) to afford the phosphonic acid terminated dendrimer (^{31}P-{^{1}H} NMR (D_2O/THFd8, 81.0 MHz): δ= 11.1 (s, PO_3H_2) ppm). The residue is suspended in water (1 mL/100 mg) in the presence of one equivalent of NaOH for one phosphonic end. The solution is filtered on microfilter (0.2 µm) and freeze-dried to afford the dendrimer with sodium salt phosphonic acid ends **7b-G$_1$** as a white solid (yield: 71%).

^{31}P-{^{1}H} NMR (D_2O/CD_3COCD_3, 81.0 MHz): δ = 10.2 ppm. ^{1}H NMR (D_2O/THFd8, 250.0 MHz): δ = 2.11 (br s, 8H, Hg); 2.41 (br s, 8H, Hf); 2.67 (br s, 8H, Hc); 3.18 (br s, 12H, Ha, Hb); 3.34 (br s, 16H, Hd, He); 3.47 (br d, $^{2}J_{HP}$ = 10.2 Hz, 24H, CH_2P, Hh); 7.37-7.77 (m, 4H, NH) ppm. ^{13}C-{^{1}H} NMR (D_2O/CD_3COCD_3, 62.9 MHz): δ = 22.8 (s, Cg); 33.4 (s, Cc); 35.2 (s, Cf); 41.3 (s, Ce); 41.4 (s, Cd); 51.3 (s, Cb); 51.7 (s, Ca); 55.1 (d, $^{1}J_{CP}$ = 130.0 Hz, CH_2P); 58.9 (br s, Ch); 175.9 (s, CONH); 177.5 (s, CONH) ppm.

Synthesis and characterization of dendrimer 7b-G_2

First step

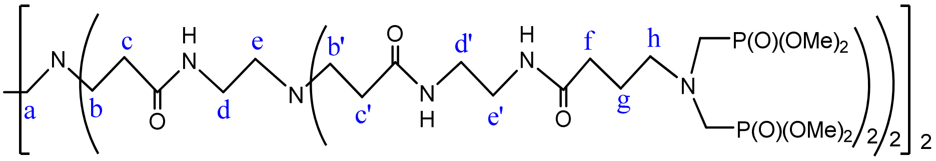

To a solution of aza-bisphosphonate carboxylic acid **12b** (x = 3; 2 mmol) in 4 mL of dry DMF at 0°C is added HOBt (2.6 mmol). The mixture is stirred at 0°C for 30 minutes, and DCC (2.6 mmol) is added. After 30 minutes at 0°C the mixture is allowed to warm to room temperature and stirring is continued for another hour, the formation of a precipitate is observed. The suspension is cooled to 0°C then second-generation PAMAM dendrimer (0.17 mmol) is added. After 30 minutes at 0°C stirring is continued at room temperature for 20 h. The precipitate is eliminated on 5 µ Millipore filters and the clear solution is freeze-dried. The product is treated three times by dissolution in a minimum volume of dichloromethane and precipitation in a large volume of diethylether so as to eliminate the excess of reagents. The dendrimer is obtained as an off-white powder (yield: 78%) after purification by column chromatography on silica gel.

^{31}P-{^{1}H} NMR ($CDCl_3$, 81.0 MHz): δ = 30.4 ppm. ^{1}H NMR ($CDCl_3$, 200.1 MHz): δ = 1.69 (br t, $^{3}J_{HH}$ = 5.9 Hz, 16H, Hg); 2.17 (br t, $^{3}J_{HH}$ = 5.9 Hz, 16H, Hf); 2.21-2.93 (m, 76H, Ha, Hb, Hc, He, Hh, Hb', Hc'); 3.06 (d, $^{2}J_{HP}$ = 9.1 Hz, 32H, CH_2P); 3.25 (br s, 40H, Hd, Hd', He'); 3.69 (d, $^{3}J_{HP}$ = 10.5 Hz, 96H, OMe); 7.70 (m, 8H, CONH); 8.07 (br s, 8H, CONH); 8.28 (br s, 4H, CONH) ppm. ^{13}C-{^{1}H} NMR ($CDCl_3$, 62.9 MHz): δ = 23.7 (s, Cg); 31.4 (s, Cc); 33.0 (s, Cc'); 33.5 (s, Cf); 37.1 (s, Cd, Ce); 39.9 (s, Cd', Ce'); 48.4 (s, Cb); 49.6 (dd, $^{1}J_{CP}$ = 158.1 Hz, $^{3}J_{CP}$ = 7.0 Hz, CH_2P); 50.4 (s, Ca, Cb'); 53.0 (br s, OMe); 56.3 (t, $^{3}J_{CP}$ = 8.2 Hz, Ch); 171.6 (s, CONH); 172.7 (s, CONH); 174.1 (s, CONH) ppm.

Second step

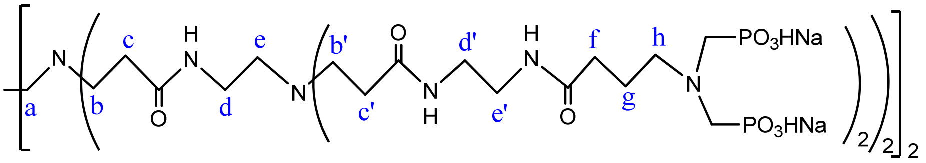

To a solution of generation 2 PAMAM-type dendrimer with azabis-phosphonate ends (0.2 mmol) as prepared above in 4 mL of freshly distilled acetonitrile at 0°C is added dropwise BrTMS (9.6 mmol). The mixture is stirred at 0°C for 30 minutes then at room temperature for

another 15 hours. The solution is evaporated to dryness under reduced pressure. The residue is treated with methanol (2 x 15 mL), washed with ether (20 mL) and finally twice with a THF/ diethylether mixture (1:9) to afford the phosphonic acid terminated dendrimer ($^{31}P-\{^1H\}$ NMR (D_2O/ CD_3COCD_3, 81.0 MHz): δ = 11.3 (s, PO_3H_2) ppm). The residue is suspended in water (1 mL/100 mg) in the presence of one equivalent of NaOH for one phosphonic end. The solution is filtered on microfilter (0.2 μm) and freeze-dried to afford the dendrimer with sodium salt phosphonic acid ends **7b-G$_2$** as a white solid (yield: 67%).

$^{31}P-\{^1H\}$ NMR (D_2O/CD_3COCD_3, 81.0 MHz): δ = 10.1 (s, PO_3HNa) ppm. ^1H NMR (D_2O/THFd8, 250.1 MHz): δ = 2.10 (br s, 16H, Hf); 2.18 (br s, 16H, Hg); 2.71 (br s, 8H, Hc); 2.80 (br s, 16H, Hc'); 3.20 (br s, 12H, Ha, Hb); 3.33 (br s, 40H, Hd; Hd', He'); 3.48 (br s, 72H, He, Hb', Hh, CH_2P); 4.41-7.79 (m, 20H, NH) ppm. $^{13}C-\{^1H\}$ NMR (D_2O/CD_3COCD_3, 62.9 MHz): δ = 22.8 (s, Cg); 32.0 (s, Cc, Cc'); 35.2 (s, Cf); 41.3 (s, Cd or Cd'); 41.6 (s, Ce, Ce'); 51.1 (s, Ca, Cb); 52.4 (s, Cb'); 55.3 (d, $^1J_{CP}$ = 132.6 Hz, CH_2P); 58.9 (s, Ch); 174.7 (s, CONH); 176.5 (s, CONH); 177.7 (s, CONH) ppm.

Synthesis and characterization of dendrimer 8a-G$_2$

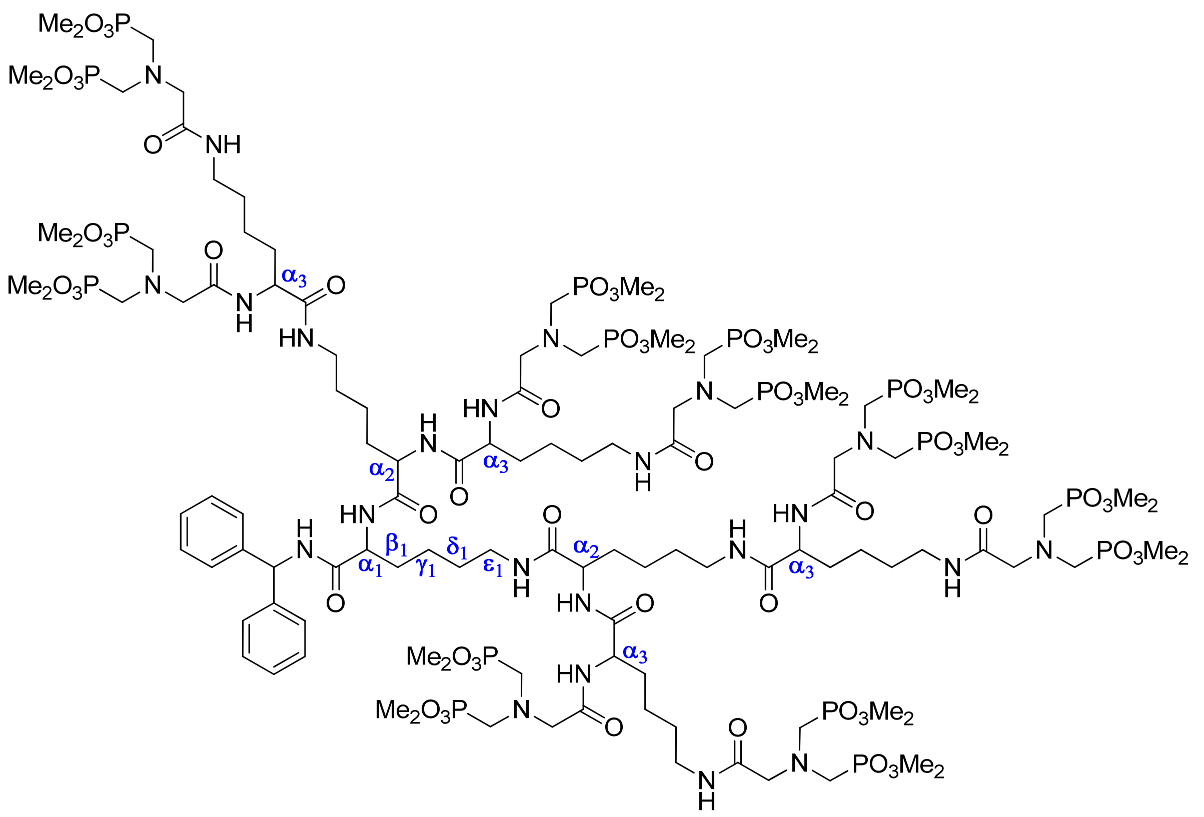

To a solution of generation 2 BHA-Lysine-100% BOC dendrimer (100 mg) in 13 mL of dichloromethane are added 7 mL of trifluoroacetic acid, and the reaction mixture is stirred for

three hours at 25°C. After evaporation of the volatiles, the crude product is dried overnight under reduced pressure, and the dry residue is solubilized in 1 mL of dry DMF at 0°C in the presence of triethylamine (8 eq.). In another flask is prepared a solution of aza-bis-phosphonate carboxylic acid **12a** (x = 1, 8.8 eq.) in 5 mL of dry DMF at 0°C and 1-hydroxybenzotriazole (8.8 eq.). This mixture is stirred for 15 minutes at 0°C then 1,3-dicyclohexylcarbodiimide (8.8 eq.) is added and the mixture is stirred for 30 minutes at 0°C then for 1 h at room temperature. The formation of a white precipitate is observed. The deprotected first generation poly-L-lysine dendrimer in DMF solution (1 mL) is then added at 0°C to the activated ester solution maintained at 0°C. Stirring is continued for 15 minutes at 0°C then overnight at room temperature. The precipitate is eliminated by filtration on a 5µ Millipore syringe filter then the solution is freeze-dried. The product is purified by dissolution in a minimum volume of CH_2Cl_2 and precipitation in a large volume of diethylether. The product is filtered on a pad of silica gel and engaged in the next step.

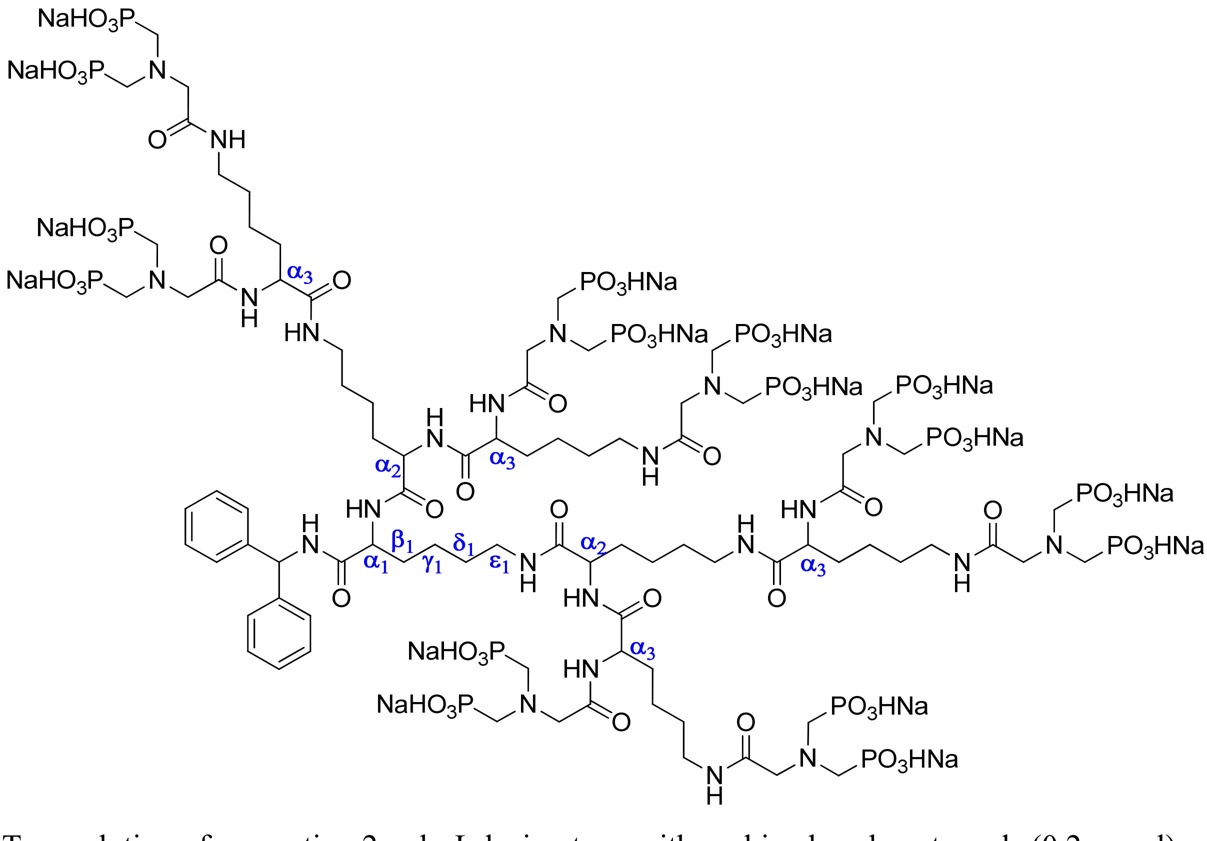

To a solution of generation 2 poly-L-lysine-type with azabis-phosphonate ends (0.2 mmol) as prepared above in 4 mL of freshly distilled acetonitrile at 0°C is added dropwise BrTMS (9.6 mmol). The mixture is stirred at 0°C for 30 minutes then at room temperature for another 15 hours. The solution is evaporated to dryness under reduced pressure. The residue is treated with methanol (2 x 15 mL), washed with ether (20 mL) and finally twice with a THF/ diethylether mixture (1:9) to afford the phosphonic acid terminated dendrimer. The residue is

suspended in water (1 mL/100 mg) in the presence of one equivalent of NaOH for one phosphonic end. The solution is filtered on microfilter (0.2 µm) and freeze-dried to afford the dendrimer with sodium salt phosphonic acid ends **8a-G$_2$** as a white solid (yield: 86%).
^{31}P-{^{1}H} NMR (D_2O/ CD_3COCD_3, 81.0 MHz): δ= 10.4 (s, PO_3HNa); 11.9 (s, PO_3HNa) ppm ^{1}H NMR (D_2O/DMSO*d6*, 200.1 MHz): δ = 1.40-1.94 (m, 42H, Hβ,γ,ó); 3.05-4.49 (m, 95H, CH_2P, Hε, $COCH_2N$, Hα); 6.15 (br s, 1H, Ph-CH); 7.45 (br s, 10H, C_6H_5) ppm. ^{13}C-{^{1}H} NMR (D_2O/DMSO*d6*/CD_3CN, 50.3 MHz): δ = 24.0-33.6 (m, Cβ,γ,ó); 45.7 (s, Cε); 47.9 (s, Cε); 51.8 (s, Cε); 54.5-56.7 (m, Cα, CH_2P); 59.7, (br s, $CHPh_2$, $\underline{CH_2}$-N-CH_2P); 129.9-131.8 (m, C_6H_5); 142.5 (m, CO); 167.8-175.9 (m, CO) ppm.

Supplementary Methods for Molecular Modeling

The MD simulation work was conducted by using the AMBER 12 software[6]. The molecular models for all dendrimers were created according to a validated procedure for the simulation of dendrimers in aqueous solution[7,8,9], and parametrized using the “general AMBER force field (GAFF)” (*gaff.dat*)[10]. The force field parameters for the **4-G$_1$** carbosilane dendrimer were obtained as previously reported[11]. All the dendrimer models were immerged in a periodic box containing explicit TIP3P water molecules[12] and a suitable number of counter-ions to guarantee the overall neutrality of the systems and to reproduce the ionic strength of NaCl (150 mM) by using the *leap* module of AMBER 12. The main features of all simulated systems are reported in Supplementary Table 1.

All systems were initially minimized and then heated for 50 ps of NVT MD simulation to reach the experimental temperature of 37 °C (310 K). During this step the solute was maintained as fixed. All restraints were then removed and the systems were equilibrated for 200 ns of NPT (constant number of N: atoms, P. pressure and T: temperature in the system) MD simulations at the experimental temperature of 37°C and 1 atm of pressure under periodic boundary conditions. The root mean square deviation (RMSD) and dendrimer size (*i.e.*, the radius of gyration – R_g) data were extracted from the simulations with the *ptraj* module of

AMBER 12 and used to check the systems equilibrium (see Supplementary Figure 1). A time step of 2 femtoseconds was adopted for all runs, as well as the Langevin thermostat and a 8 Å cutoff. The long-range electrostatic effects were treated according to the particle mesh Ewald (PME) approach[13]. The SHAKE algorithm was used on all bonds involving Hydrogen atoms[14]. All data analyses were performed on the equilibrated phase MD trajectories – *i.e.*, the last 50 ns of each MD run. The structural analyses (calculation of R_g, *g(r)* plots, aspect ratios, anisotropy and SASA (Supplementary Figure 2), etc.) and the analysis on the hydration levels of the dendrimers (Supplementary Figure 3) were conducted by using the *ptraj* module of AMBER 12. In particular, the aspect ratio data (Supplementary Figure 2c) were calculated from the principal moments of inertia (being $I_z > I_y > I_x$) of the equilibrated dendrimers[15,16]. Similarly, the shape anisotropy parameter (Supplementary Figure 2: κ^2, in black) was calculated as: $\kappa^2 = 1 - 3(\langle I_2\rangle/\langle I_1^2\rangle)$, being $I_1 = I_x + I_y + I_z$ and $I_2 = I_xI_y + I_yI_z + I_xI_z$[15,16].
The solvation energies G_{sol} were calculated directly from the equilibrated phase MD simulations of each dendrimer according to the MM-GBSA approach[17,18]. In particular, G_{sol} was calculated as: $G_{sol} = G_{GB} + G_{NP}$[19]. The polar component of solvation (G_{GB}) was evaluated according to the generalized Born approach[20,21]. The non-polar term of the solvation energy was calculated as $G_{NP} = g\ (SASA) + b$, where $g = 0.00542$ kcal/Å^2, $b = 0.92$ kcal/mol, and SASA is the solvent-accessible surface estimated with the MSMS program (Supplementary Figure 2d)[22].

Supplementary References

1 Griffe, L. et al. *Angew. Chem. Int. Ed.* **46,** 2523–2526 (2007).

2 Salamonczyk, G. M., Kuznikowski, M. & Skowronska, A. *Tetrahedron Lett.* **41,** 1643–1645 (2000).

[3] de Groot, D., Reek, J. N. H., Kamer, P. C. J. & van Leeuwen, P. W. N. M. *Eur. J. Org. Chem.* **6,** 1085–1095 (2002).

[4] Launay, N., Caminade, A. M. & Majoral, J. P. *J. Organomet. Chem.* **529,** 51–58 (1997).

[5] Ornelas, C., Mery, D., Blais, J. C., Cloutet, E., Aranzaes, J. R. & Astruc, D. *Angew. Chem. Int. Ed.* **44,** 7399–7404 (2005).

[6] Case, D. A. et al. AMBER 12. In University of California, San Francisco (2012).

[7] Pavan, G. M., Barducci, A., Albertazzi, L. & Parrinello, M. *Soft Matter* **9,** 2593–2597 (2013).

[8] Garzoni, M., Okuro, K., Ishii, N., Aida, T. & Pavan, G. M. *ACS Nano* **8,** 904–914 (2014).

[9] Simanek, E. E., Enciso, A. E. & Pavan, G. M. *Exp. Opin. Drug Disc*. **8,** 1057–1069 (2013).

[10] Wang, J., Wolf, R. M., Caldwell, J. W., Kollman, P. A. & Case, D. A. *J. Comput. Chem.* **25,** 1157–1174 (2004).

[11] Fuentes-Paniagua, E. et al. *RSC Advances*, **4,** 1256–1265 (2014).

[12] Jorgensen, W. L., Chandrasekhar, J., Madura, J. D., Impey, R. W. & Klein, M. L. *J. Chem. Phys.* **79,** 926–935 (1983).

[13] Darden, T., York, D. & Pedersen, L. *J. Chem. Phys.* **98,** 10089–10092 (1993).

[14] Krautler, V., van Gunsteren, W. F. & Hunenberger, P. H. *J. Comput. Chem*. **22,** 501–508 (2001).

[15] Maiti, P. K., Cagin, T., Wang, W. & Goddard, W. A. *Macromolecules* **37,** 6236–6254 (2004).

[16] Furlan, S., La Penna, G., Appelhans, D., Cangiotti, M., Ottaviani, M. F. & Danani, A. *J. Phys. Chem. B* **118,** 12098–12111 (2014).

[17] Kollman, P. A., et al. *Accounts Chem. Res.* **33,** 889–897 (2000.

[18] Srinivasan, J., Cheatham, T. E., Cieplak, P., Kollman, P. A. & Case, D. A. *J. Am. Chem. Soc.* **120,** 9401–9409 (1998).

[19] Jayaram, B., Sprous, D. & Beveridge, D. L. *J. Phys. Chem.* **102,** 9571–9576 (1998).

[20] Onufriev, A., Bashford, D. & Case, D. A. *Proteins*, **55,** 383–394 (2004).

[21] Onufriev, A., Bashford, D. & Case, D. A. *J. Phys. Chem. B*, **104,** 3712–3720 (2000).

[22] Sanner, M. F., Olson, A. J. & Spehner, J. C. *Biopolymers* **38,** 305–320 (1996).
